# Effects of complementary feeding on attained height among lower primary school-aged children in Eastern Uganda: A nested prospective cohort study

**DOI:** 10.1371/journal.pone.0211411

**Published:** 2019-02-07

**Authors:** Espoir Bwenge Malembaka, James K. Tumwine, Grace Ndeezi, Ingunn Marie Stadskleiv Engebretsen, Thorkild Tylleskär, Henry Wamani, Halvor Sommerfelt, Victoria Nankabirwa

**Affiliations:** 1 Ecole Régionale de Santé Publique, ERSP, Faculté de Médecine, Université Catholique de Bukavu, Bukavu, Democratic Republic of Congo; 2 Institute of Health and Society, IRSS, Université Catholique de Louvain, Brussels, Belgium; 3 Department of Epidemiology and Biostatics, School of Public Health, College of Health Sciences, Makerere University, Kampala, Uganda; 4 Department of Paediatrics and Child Health, School of Medicine, College of Health Sciences, Makerere University, Kampala, Uganda; 5 Global Mental Health Research Group, GMHRG, Centre for International Health, University of Bergen, Bergen, Norway; 6 Centre for Intervention Science in Maternal and Child Health, Centre for International Health, University of Bergen, Bergen, Norway; 7 Department of Community Health and Behavioral Sciences, School of Public Health, College of Health Sciences, Makerere University, Kampala, Uganda; Medical Research Council, SOUTH AFRICA

## Abstract

**Background:**

Despite the fact that Uganda has been a signatory to the global strategy for Infant and Young Children Feeding practices (IYCF) for nearly a decade, the prevalence of stunting among children under five years of age remains tragically high at 17% in Eastern Uganda and twofold higher countrywide. Only 6% of all children aged 6–23 months feed adequately. This study aimed to establish the covariates of complementary feeding (CF) and its effect on attained height among primary school-aged children in Mbale district (Eastern Uganda).

**Methods:**

This was a community-based prospective cohort study using data from the PROMISE EBF trial. The main exposure variable was adequate complementary feeding (CF) measured in a parent questionnaire at 18–24 months of age. We defined adequate CF as having received animal food, cereals and fruit, juice and/or vegetables during the 24 hours preceding the interview. An adapted minimum acceptable diet was defined as having been given milk or milk products at least twice a day, an adapted meal frequency of two and solid or semi-solid food from at least four food groups on a 24-hour dietary recall based on modified IYCF criteria. The main outcome variable was attained height [(height-for-age Z score (HAZ)] measured between five and eight years of age using the WHO growth standards. Effects of CF on HAZ were estimated using linear regression analyses with cluster-robust standard errors.

**Results:**

A total of 506 children were studied. The majority (85%) were from rural areas and the average age at the end of the study was 6.9 (standard deviation: 0.63) years. Of these, 23.9% were adequately fed and 2.3% received the adapted minimum acceptable diet. Adequate CF was not associated with HAZ (adjusted β = -0.111; 95% CI: -0.363, 0.141; p = 0.374). Factors significantly associated with attained height were baseline HAZ (0.262; 0.152, 0.374; p<0.001) and WHZ (-0.147; -0.243, -0.051; p = 0.004), child’s age (0.454; -0.592, -0.315; p<0.001) and maternal education (0.030; 95% CI: 0.003, 0.057; p = 0.034).

**Conclusion:**

Adequate CF at age 18–24 months was worryingly insufficient and not associated with attained HAZ at age 5–8 years. Further strategies need to be considered to improve child nutrition and linear growth in resource-constrained settings.

## Introduction and background

Globally, stunting, which is a reflection of chronic undernutrition affects nearly 1 in every 4 children less than five years of age [1]. Linear growth is recognized as the best overall marker of a child’s well-being and there is convincing evidence associating stunting with high child mortality, increased susceptibility to infection and poor cognitive and psychomotor development [2–4]. Unlike other regions of the world, sub-Saharan Africa has registered a 23% increase in the prevalence of stunting over the last two decades, and it harbours one-third of all stunted children [1]. This is partially explained by population growth, insufficient economic development and programmatic strategies to improve the nutritional situation for children [5]). In Uganda, the 2016 Demographic and Health Survey indicated that the prevalence of stunting remains high, varying from 11.8% among children under 6 months of age to 36.8% among those aged between 24–35 months, with an overall prevalence of 29% under 5 years of age [6]. Previous cross-sectional studies from Eastern Uganda found stunting to be significantly associated with mixed feeding in the first six months of life, against a background of poverty and food insecurity [7, 8].

The link between child feeding practices including inadequate complementary feeding (CF) and malnutrition is no longer debatable. CF for infants refers to the timely introduction of safe and nutritionally rich foods in addition to breast-feeding at about 6 months of age and is typically provided from 6 to 23 months of age [9]. Thus, it coincides with and impacts on a critical period of linear growth and cognitive development. Complementary foods are necessary in early childhood to fill the constantly increasing gap between the total nutritional needs of the child and the amount of nutrients provided by breast milk. While adequate and appropriate complementary feeding is one of the key evidence-based interventions with a potential to noticeably reverse the prevalence of undernutrition in low income countries [10], it has drawn relatively little attention in comparison to breast and formula feeding which have been widely studied. Numerous studies indicate that in-spite of optimal breastfeeding, quantitatively and qualitatively inadequate complementary feeding from six months of age onwards could lead to stunting [11].

While adequate CF can potentially bring about a one-third decline in the prevalence of stunting in children less-than 36 months of age, avert about one-quarter of child deaths among these children and prevent up-to 60 million DALYs [10], its effects on attained growth of primary school-aged children has not been evaluated adequately in Uganda or in other sub-Saharan countries. In addition, limited information is available on various dietary compositions and their effect on long-term growth. Context specific information is needed as diets vary with culture and regions. The few descriptive studies of CF in developing countries are cross-sectional, with relatively small sample sizes, and were in most cases done in urban areas [12]. We used existing food recalls with known dietary compositions gathered in a systematic manner to assess the long-term effect of CF on attained longitudinal growth over a 5–8 years follow-up.

## Methods

### Design

This was a community-based prospective cohort study nested in the PROMISE EBF trial (ClinicalTrials.gov.no. NCT00397150) and the follow-up PROMISE Saving Brains (SB) study in Mbale District, Eastern Uganda [13, 14]. The PROMISE-EBF study was a multicenter community-based cluster-randomised behavioural-intervention trial. The study was undertaken in three Sub-Saharan African (SSA) countries; Burkina Faso (Banfora), South Africa (Umlazi, Rietveli Paarl regions) and Uganda (Mbale district). This paper includes study participants from the Ugandan site only with respective relevant follow-up studies after 5–8 years, thus providing a relatively long prospective longitudinal cohort from this site.

### Study settings and participants

Mbale district is situated approximately 230 km east of Kampala. The study was carried out in the two biggest counties in the district, urban Mbale Municipality and rural Bungokho. In 2006, when the PROMISE EBF cohort was initiated, Mbale district had an estimated population of 403,000 inhabitants with a population density of 535 per square kilometre. Generally, subsistence agriculture along with petty trading are the main economic activities in Mbale district where nearly 75% of the population live in rural areas [15].

The study population was comprised of singleton children born to women that had an intention to breastfeed and lived in the study area between January 2006 and May 2008. Women who were at least 7 months or visibility pregnant were identified by community-based recruiters and pre-included in the PROMISE EBF cohort. In post-partum, mother-infant pairs were kept in the study if the newborns did not have any congenital malformation that could interfere with breastfeeding.

### Study sample and follow-up

**Fig 1:**
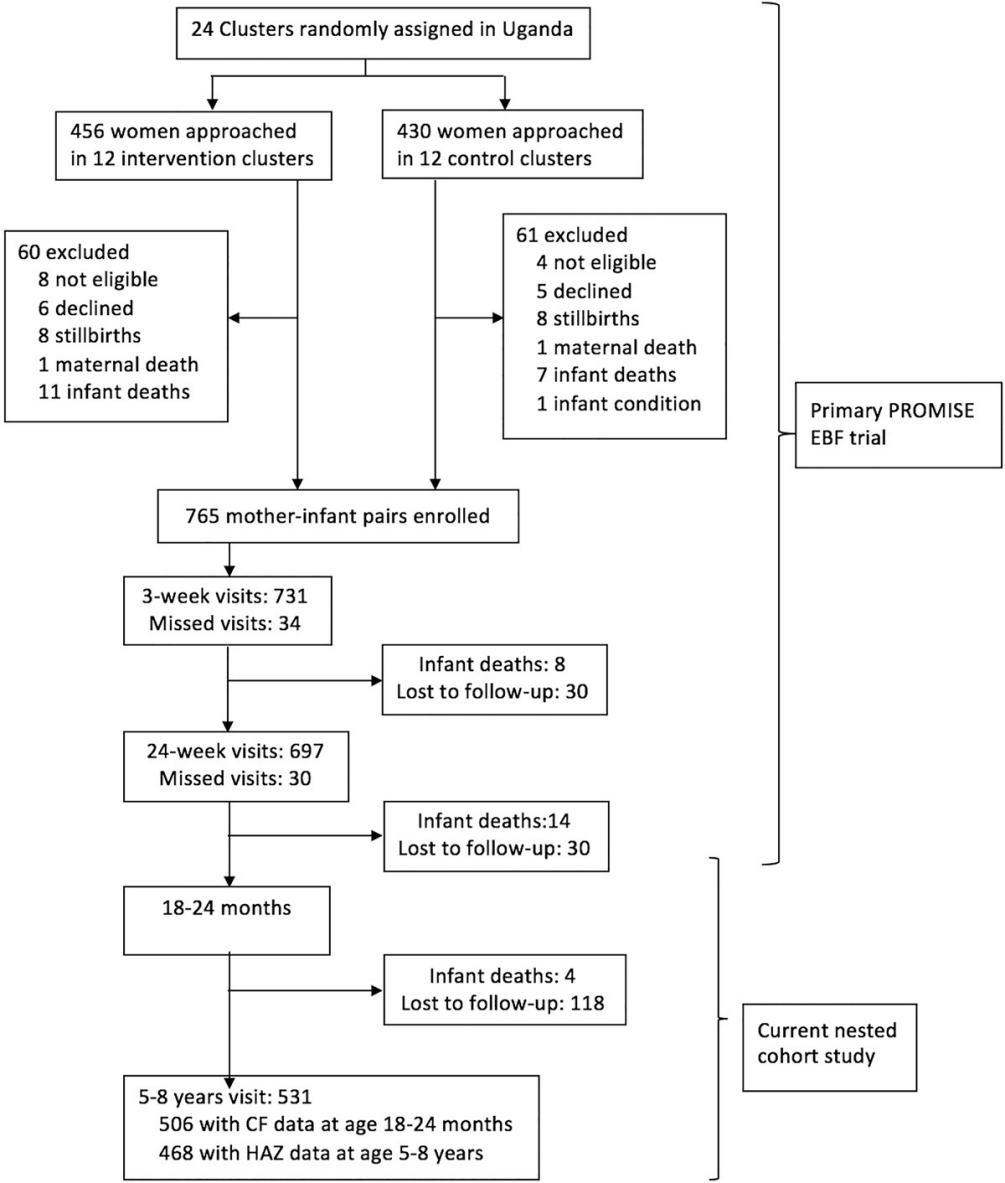
Flow diagram of the study population.

The cohort size was determined by the cluster randomized trial from which this study arose. Exclusive breastfeeding prevalence and decrease in diarrheal rate were the two outcomes on which the sample size calculation for the primary PROMISE EBF trial was based as described in detail elsewhere [13]. Out of 765 mother-infant pairs included in the original PROMISE EBF trial, 506 children had adequate CF data at 18–24 months of age and 468 children had attained height data at 5–8 years of age, after setting to missing outlying and implausible HAZ values (beyond the flag boundaries of -6 and +6 Z) (Fig 1).

### Data collection

#### Primary PROMISE EBF

Exposure data (complementary feeding) were collected when the children were aged between 18–24 months and outcome data (attained height) were collected at age 5–8 years. Data collectors fluent in both the local language (Lumasaaba) and English were recruited through newspaper advertisement. They were trained for one week at the commencement of the original PROMISE EBF trial and with refresher training 9 months later and then again before each new round of data collection. Data were collected using both electronic (18–24 months) and paper-based (5–8 years) structured pre-coded questionnaires. Electronic data were captured on hand-held computers with the Epi Handy tool (version 165.528–142 RC). Data on background characteristics of the participants were collected during pregnancy in order to describe the study population and to identify potential confounders. Follow-up questionnaires were used to collect information on complementary feeding and growth.

#### Current analyses

Baseline data for the present nested study were collected when the children were aged 18–24 months. Follow up data collection was carried out between 2013 and 2015 when the children were 5–8 years old. At 18–24 months and 5-8-year time points, anthropometric data were collected following standard WHO guidelines (http://www.who.int/childgrowth/en/). Infant length between 18–24 months of age was measured to the nearest 0.1 cm using the “*Baby/infant/adult Length-height measuring system SET 2”*and undressed children were weighed to the nearest 0.1kg using the *“Infant scale spring type*, *25 kg*, *100 g*” from UNICEF. Between 5–8 years of age, a Seca stadiometer® was used to measure height and weight was measured using digital Seca scales to the nearest 0.1 kg.

### Study variables

The main explanatory variable was adequate CF, a dichotomous variable based on a 24-hour structured dietary recall to a locally adapted food item list at 18–24 months of age. A child was considered as having received adequate CF if they were given animal food, cereals and fruit, juice and/or vegetables during the 24 hours preceding the interview. The 24-hour dietary recall consisted in asking mothers or caregivers about the food items children were given during the 24 hours preceding the interview, formulated as “from yesterday morning till this morning”. We also examined child feeding at 5–8 years of age based on a 7-day dietary recall whereby questions were asked about the same food items as for the 24-hour recall. Additionally, we used slightly modified IYCF criteria based on breastfeeding status of children aged 6–23 months [16]. Here, a child was considered as having received an adapted minimum acceptable diet if they were given milk or milk products at least twice a day, an adapted minimum meal frequency and solid or semi-solid food from at least four food groups. The modification of the IYCF criteria lay in the definition of the adapted minimum meal frequency that was referred to as having received solid or semi-solid food at least twice a day, regardless of the age of the child and his or her breastfeeding status. The original definition recommends a minimum frequency of 2 times for breastfed infants aged 6–8 months, 3 times for breastfed children between 9–23 months of age and 4 times for non-breastfed children aged 6–23 months. Given the extremely low prevalence of adequate CF in this population, we opted for the modified and much more contextualized IYCF criteria.

At 5–8 years of age, adequate child feeding definition was based on a seven-day structured dietary recall. The following are the main food groups of which a minimum of four is required along with other IYCF practices for adequate feeding: infant formula, milk other than breast milk, cheese or yogurt or other milk products; foods made from grains, roots, and tubers, including porridge and fortified baby food from grains; vitamin A-rich fruits and vegetables, other fruits and vegetables, eggs, meat, poultry, fish, and shellfish (and organ meats); legumes and nuts.

The study outcome was HAZ reflecting attained height and defined according to the WHO Child Growth Standards [17, 18]. We classified a child as stunted if she or he had an HAZ score below -2 SD. We also report descriptive data for weight-age Z score, weight-for-height Z score and BMI-for-age Z score.

Potential confounders encompassed socio-demographic characteristics, including socio-economic status (SES), and breastfeeding status at 24 weeks and at 18–24 months of age. A wealth index at baseline was created as a proxy for socio-economic status of the household using Multiple Correspondence Analysis (MCA) from a set of wealth items and housing characteristics, including number of rooms and beds, roof material, ownership of bicycle, motor vehicles, radio, television, lantern, cupboard, and source of drinking water. Three SES levels were generated to rank the families of the children and divided in quintiles, a strategy being widely used in Demographic and Health Surveys in low- and middle-income countries. Wealth status groups were then made of the lowest quintile (20%), the mid two quintiles (40%) and the top two quintiles (40%) which was believed be a realistic presentation of the wealth distribution in this context. Exclusive breastfeeding under 24 weeks was measured at the 24-week visit and defined as not having received any other food or liquids other than breast milk based on seven-day recall [13] Breastfeeding at 18–24 months was a binary variable assessing whether a child was still breastfeeding at the 18–24 months visit.

### Statistical analyses

For this study, we included all children with anthropometric data between 5 and 8 years of age. Anthropometric indices were generated using WHO AnthroPlus 2009 software http://www.who.int/growthref/tools/en/. Data were analysed with the use of Stata SE 14.0 (Stata Corp LP, College Station, TX, USA). For the crude analysis, descriptive statistics included means and their standard deviations (SD) as well as medians with interquartile ranges (IQR) for continuous variables. Categorical variables were summarized into frequencies and proportions. To examine the association between the contextualized adequate CF and attained height we fitted bivariable and multivariable linear regression models, using clustered robust standard errors to take into account the cluster design of the primary PROMISE EBF study. Variables were included in multiple regression models when they were associated with complementary feeding and the outcomes at a p-value of ≤ 0.2 or based on biological plausibility. We examined the distribution of continuous variables with respect to symmetry and heteroscedasticity (i.e. the degree to which variance is different between the children with adequate as compared to those without adequate CF) using scatter plots. Normality and homoscedasticity were tested for using the Shapiro-Wilk and Breusch-Pagan tests, respectively. Multicollinearity between explanatory variables was assessed using the variance inflation factor. A sensitivity analysis based on inverse-probability weighting was conducted to assess whether the loss to follow-up between 18–24 months and 5-8-year time points influenced the effect of adequate CF on attained height. We used a logit model to estimate the average treatment-effect. The significance level for all the analyses was set to α = 5%.

### Ethical considerations

The study was approved by the Makerere University School of Public Institutional Review Board, the Makerere University Research and Ethics Committee (REC. Ref. 2012–177) and the Uganda National Council for Science and Technology (Ref. SS 3123). All the pregnant women enrolled in the PROMISE EBF trial had full authority to consent for themselves for participation in the study and provided a written informed consent. A thumb-printed signature was obtained from each caretaker.

## Results

### General characteristics of the study participants

Of 765 mother-infant pairs enrolled in the PROMISE EBF study in Uganda, 653 (85.4%) were visited at 18–24 months of age, of which 531 (81.3%) were traced and 506 (77.5) had HAZ data at 5–8 years of age. The average age of children was 17.6 (SD: 0.78) months and the range was 18–24 months when CF (exposure data) and baseline growth data were collected and 6.9 (SD: 0.63) years with a range of 5–8 years when follow-up growth data (outcome data) were collected. The mean age and years of education of mothers at the time of recruitment was 26.1 (SD: 6.6) and 6.1 (SD: 3.2) years respectively. Over 85% (430/505) of the participants lived in rural areas during late pregnancy (Table 1). The majority of mothers [81.3% (408/502)] were multipara and 6% (29/502) were not married at recruitment at the commencement of the PROMISE EBF study. Up to 22.8% (115/505) of the participants were living in poorest households and 35.8% (181/505) in least poor ones. About 67.1% (337/502) of the participants had no electricity in their homes and 30.2% (152/505) were using surface water sources. Twenty-five percent (124/492) of children were exclusively breastfed at 24 weeks and 47.3% (239/506) were still breastfeeding at 18 months of age, based on seven-day recall. Up to 24.4% (118/484) of mothers did not attend ANC clinics even once (Table 1).

**Table 1:** General characteristics of the study participants.

Characteristics	Summary statistics
Categorical data	
Residence	505
Urban	75 (14.9%)
Rural	430 (85.1%)
Marital status of the parents	502
Married	337 (67.1%)
Cohabiting	136 (27.1%)
Single/widowed/divorced	29 (5.8%)
Wealth status	505
Poor	115 (22.8%)
Middle	209 (41.4%)
Least poor	181 (35.8%)
Exclusive breastfeeding at 24 weeks	492
Yes	124 (25.2%)
No	368 (74.8%)
Breastfeeding at 18–24 months	506
Yes	239 (47.2%)
No	267 (52.8%)
Parity of the mother	502
Primipara	94 (18.7%)
Multipara	408 (81.3%)
Electricity at home	502
Yes	165 (32.9%)
No	337 (67.1%)
Water source	504
Surface water or other	175 (34.7%)
Borehole or tap	298 (59.1%)
Piped into yard or home	31 (6.2%)
ANC attendance	484
Yes	366 (75.6%)
No	118 (24.4%)
Continuous data	
Mother’s age	498
Mean (SD)	26.1 (6.6)
Median (IQR)	25 (21–3)
Child’s age, 5–8 years visit	479
Mean	6.9 (0.6)
Median	6.9 (6.4–7.4)
Mother’s education (years)	500
Mean (years)	6.1 (3.2)
Median (IQR)	6 (4–8)
Father’s education (years)	461
Mean (SD)	7.6 (3.47)
Median (IQR)	7 (5–11)

Data are number, number (%), mean (SD) and median (IQR); they were collected late in pregnancy.

### Child feeding patterns

#### Infant and young child feeding characteristics in Mbale district

The proportion of children that received the adapted minimum acceptable diet at 18–24 months of age was 0.27% based on the strict IYCF criteria and 2.32% when using the modified IYCF definition. Approximately 30% (150/506) of children were given animal food whereas 0.4% (2/506) received food from all four food groups during the 24 hours prior to the interview (Table 2). About three-quarters of the children were fed cereals and less than half were given fruits, juice and or vegetables (Table 2). Considering the seven-day dietary recall, up to 61.9% (313/506) of the children were given animal food at 18–24 months of age against 19.4% (98/504) at 5–8 years. The percentage of children receiving fruits, juice and or vegetables during the last 7 days before the interview more than halved, from 68.2% (345/506) to 33.6% (169/503) between the 18–24 months and 5–8 years of age. The number of children receiving milk products also dropped between the two follow-ups, from 54.6% (276/506) to 14.9% (75/504) (Table 2).

**Table 2:** Child feeding characteristics in Mbale district.

Indicator	Yes	No	Total
	N (%)	N (%)	N (%)
24h dietary recall,18–24 months of age			
Animal food	150 (29.6)	356 (70.4)	506 (100)
Cereals	368 (72.7)	138(27.3)	506 (100)
Food from 4 food groups	2 (0.4)	504 (99.6)	506 (100)
Fruits, juice and/or vegetables	250 (49.4)	256 (50.6)	506 (100)
Meal frequency			
≥3	15 (3.6)	401 (96.4)	416 (100)
≥2	153 (36.8)	263 (63.2)	416 (100)
Milk or milk product			
At least once	160 (31.6)	346 (68.4)	506 (100)
At least twice	125 (24.7)	381 (75.3)	506 (100)
Adapted minimum acceptable diet*	13 (2.3)	547 (97.7)	506 (100)
Adequate CF **	121(23.9)	385 (76.1)	506 (100)
7-day dietary recall, 18–24 months of age			
Animal food	313 (61.9)	193 (38.1)	506 (100)
Cereals	426 (84.2)	80 (15.8)	506 (100)
Fruits, juice and/or vegetables	345 (68.2)	161 (31.8)	506 (100)
Milk or milk products	276 (54.6)	230 (45.4)	506 (100)
CF with 3 food groups**	121(23.9)	385 (76.1)	506 (100)
7-day dietary recall, 5–8 years of age			
Animal food	98 (19.4)	406 (80.6)	504 (100)
Cereals	399 (79.2)	105 (20.8)	504 (100)
Fruits, juice and/or vegetables	169 (33.6)	334 (66.4)	503 (100)
Milk of milk products	75(14.9)	429 (85.1)	504 (100)
Adequate child feeding**	57 (11.3)	446 (88.7)	503 (100)

CF: complementary feeding

*Adapted minimum acceptable diet defined as a combination of having received animal food, fruit, juice or vegetables, cereals and milk or milk products, having been fed at least twice the last 24 hours in addition to being given milk or milk products.

**Adequate child feeding with three food groups: animal food, cereals and fruits, juice and/or vegetables.

When using the relaxed definition of adequate CF much more adapted to the context of rural Uganda and whereby a child was adequately fed if they were given animal food, cereals and fruits, juice and/or vegetables during the last 24 hours, we found that 23.9% (121/506) qualified as being adequately fed between 18–24 months of age against 11.33% (57/503) between 5–8 years of age.

#### Characteristics of the study population by complementary feeding status

The results on the characteristics of the study cohort by child complementary feeding status at age 18–24 months are presented in Table 3. Of the 384 (76.0%) children inadequately fed, the proportion of rural area residents (78.6%) was significantly higher than that of urban area residents (p = 0.001). At age 5–8 years, the mean age of children who received adequate CF between 18–24 months of age [6.74 (SD: 0.60)] was lower than that [6.97 (SD: 0.63)] of children who received inadequate CF (<0.001). There was no association between CF status with mother’s age, marital status, years of education, wealth status, EBF status, breastfeeding status between 18–24 months, parity, having electricity in house, water source and ANC attendance in bivariable analysis (Table 3).

**Table 3:** Characteristics of the study population by complementary feeding status at 18–24 months of age.

Characteristics	Adequate CF N (%)	Inadequate CF N (%)	P-value
Categorical variables			
Residence	121 (24.0)	384 (76.0)	<0.001
Urban	29 (38.7)	46 (61.3)	
Rural	92 (21.4)	338 (78.6)	
Marital status of the parents	119 (23.71)	383 (76.29)	0.048
Married	70 (20.7)	267 (79.3)	
Cohabiting	42 (30.9)	94 (69.1)	
Not in union	7 (24.1)	22 (75.9)	
Wealth status	121 (24.0)	384 (76.0)	0.280
Poor	27(23.5)	88 (76.5)	
Middle	43 (20.6)	166 (79.4)	
Least poor	51(28.2)	130 (71.8)	
Exclusive breastfeeding at 24 weeks	115 (23.4)	377 (76.6)	0.012
Yes	21 (17.0)	103 (83.0)	
No	94 (25.5)	274 (74.5)	
Breastfeeding at 18–24 months	121 (23.9)	385 (76.1)	0.995
Yes	57 (23.8)	182 (76.2)	
No	64 (24.0)	203 (76.0)	
Parity	119 (23.7)	383 (76.3)	0.031
Primipara	30 (31.9)	64 (68.1)	
Multipara	89 (21.81)	319 (78.19)	
Electricity	119 (23.7)	383 (76.3)	0.245
Yes	44 (26.7)	121 (73.3)	
No	75 (22.3)	262 (77.7)	
Water source	120 (23.8)	384 (76.2)	0.974
Surface water or other	41 (23.4)	134 (76.6)	
Borehole or tap	72 (24.2)	226 (75.8)	
Piped yard or home	7 (22.6)	24 (77.4)	
ANC attendance	114 (23.6)	370 (76.4)	0.200
Yes	92 (25.1)	274 (74.9)	
No	22 (18.6)	96 (81.4)	
Continuous variables			
Mother’s age	116 (23.3)	382 (76.7).	
Mean	25.64 (6.75)	26.25(6.50)	0.532 *
Median	24 (20–30.5)	25 (21–30)	
Child’s age, 5–8 years visit	106 (22.13)	373 (77.87)	
Mean	6.74 (0.60)	6.97 (0.63)	< 0.002
Median	6.61(6.34–7.10)	6.99 (6.40–7.44)	
Mother’s education (years)	118 (23.6)	382 (76.4)	
Mean (years)	6.64 (3.3)	5.98(3.2)	0.096
Median (IQR)	7 (5–9)	6 (4–8)	
Father’s education (years)	110 (23.9)	351 (76.1)	0.811
Mean (SD)	7.65 (3.7)	7.55 (3.4)	
Median (IQR)	7 (5–11)	7 (5–11)	

*Statistical test was run after log-transformation of the continuous variable.

Data are n (%), mean (SD), or median (IQR). The marital status “not in union” includes mothers who were single, widowed, separated, or divorced. All the data were collected during late pregnancy except for child’s age. P-values are based on cluster-robust standard errors.

### Nutritional status of children

Nearly 55% (250) of the children were stunted at 18–24 months of age and the average HAZ was -2.13 (SD: 1.12), similar in the two child feeding groups (p = 0.22) (Table 4). The mean WAZ was -1.15 (SD: 1.15) and was not statistically different by feeding status (p = 0.37). The mean WHZ was -0.34 (SD: 0.77) and it did not significantly vary by adequate CF status (p = 0.83). Overall, attained height tended to improve by 5–8 years of age with the average HAZ passing above -2 z-score, though its mean difference by child feeding status at 5–8 years of age did not reach a statistical significance (p = 0.07) (Table 4). Stunting prevalence decreased by more than 37% during follow-up from 55.95% at 18–24 months of age to 18.59% at 5–8 years of age.

**Table 4:** Nutritional outcomes at 18–24 months and 5–8 years of age by child feeding status measured at 18–24 moths and 5–8 years of age respectively.

Outcome	Adequate child feeding	Inadequate child feeding	Total	p-value
At 18–24 months of age^a^				
HAZ	n = 104	n = 349	n = 453	
Mean (SD)	-1.98 (1.18)	-2.18 (1.09)	-2.13 (1.12)	0.22
Median (IQR)	-1.82 (-2.72, -1.20)	-2.20 (-2.97, 1.47)	-2.14 (-2.92, -1.37)	
WAZ	n = 105	n = 348	n = 453	
Mean (SD)	-1.07 (1.14)	-1.18 (1.15)	-1.15 (1.15)	0.37
Median (IQR)	-1.15 (-1.77, -0.15)	-1.15 (-1.94, 0.34)	-1.16 (-1.91, -0.29)	
WHZ	n = 89	n = 276	365	
Mean (SD)	-0.32 (0.76)	-0.35 (0.77)	-0.34 (0.77)	0.83
Median (IQR)	-0.47 (-0.82, 0.13)	-0.33 (-0.82, 0.12)	-0.34 (-0.82, 1.23)	
At 5–8 years of age^b^				
HAZ	n = 46	n = 422	n = 468	
Mean (SD)	-0.75 (0.97)	-1.09 (1.11)	-1.05 (1.10)	0.07
Median (IQR)	-0.66 (-1.34, -0.20)	-1.09(-1.88, -0.37)	-1.05 (-1.78, -0.34)	
WAZ	n = 47	n = 422	n = 469	
Mean (SD)	-0.68 (0.83)	-0.92 (0.94)	-0.90 (0.93)	0.051
Median (IQR)	-0.65 (-0.98, -0.27)	-0.89 (-1.48, -0.39)	-0.87 (-1.43, -0.38)	
BAZ	n = 39	n = 402	n = 441	
Mean (SD)	-0.26(0.83)	-0.32 (0.78)	-0.32(0.78)	0.69
Median (IQR)	-0.31 (-0.91, 0.55)	-0.33 (-0.81,0.21)	-0.32 (-0.82, 0.21)	

^a^: Child feeding refers to complementary feeding.

^b^: Child feeding was defined based on a seven-day dietary recall. P-values are based on cluster-robust standard errors.

### Effect of complementary feeding on attained height (HAZ)

The key findings of the linear regression modelling for the relationship between attained length and height (HAZ) and its associated factors are reported, Table 5. Complementary feeding between 18–24 months of age appeared to have no statistically significant effect on attained height at age 5–8 years. The factors found to be positively associated with HAZ at bivariable analysis were baseline HAZ, urban residence, being least poor compared to being poorest, not breastfeeding up to 18–24 months, parental education and mother having attended at least one ANC service. In the adjusted analysis, HAZ, reflecting attained height, was significantly negatively associated with child age at the 5–8 years visit, baseline WHZ and the number of years of maternal education, and positively associated with baseline HAZ and mother’s number of years of education. (Table 5). A one-year increase in child’s age at the 5–8 years visit was associated with a decline in HAZ of 0.454 on average (95% CI: -0.592, -0.315; p<0.001), after controlling for complementary feeding status, residence, EBF status at 24 weeks of age, mother’s education, electricity in house, ANC attendance and baseline HAZ and WHZ. HAZ increased by 0.030 on average (95% CI: 0.003, 0.063; p = 0.034) for every one-year increase in maternal education, adjusting for other covariates. In the regression model without baseline HAZ and WHZ, rural residence was significantly negatively associated with attained height. In fact, the average HAZ was lower [-0.506 (95% CI: (-0.987, -0.025; p = 0.040)] in children living in rural areas compared to those in urban areas, holding the other parameters in the regression model constant (S1 Table). This suggests an intermediate role of HAZ and WHZ in the causal pathway between place of residence and attained height.

**Table 5:** Unadjusted and multivariable linear regression analyses of the association between the contextualized adequate CF at 18–24 months of age and attained height (HAZ) at age 5–8 years, with HAZ and WHZ at baseline.

Covariate	Unadjusted slope (95% CI)	P-value	Adjusted slope (95% CI)	P-value
CF				
Inadequate	Ref.		Ref.	
Adequate	0.032 (-0.261, 0.326)	0.822	-0.109 (-0.329, 0.112)	0.318
Child age at 5–8 years visit	-0.299 (-0.465, -0.132)	0.001	-0.454 (-0.592, -0.315)	<0.001
HAZ between 18–24 months	0.440 (0.326, 555)	<0.001	0.262 (0.152, 0.374)	<0.001
WHZ between 18–24 months	-0.159 (-0.280, -0.039)	0.012	-0.147 (-0.243, -0.051)	0.004
Residence				
Urban	Ref.		Ref.	
Rural	-0.670 (-1.204, -0.136)	<0.016	-0.178 (-0.462, -0.106)	0.207
Wealth status				
Poor	Ref.		Ref.	
Middle	0.095 (-0.165, 0.354)	0.458	0.043 (-0.237, 0.324)	0.753
Least poor	0.577 (0.190, 0.964)	<0.005	0.187 (-0.090, 0.463)	0.176
Mother’s education (years)	0.057 (0.0193, 0.057)	0.005	0.030 (0.003, 0.057)	0.034
EBF status				
No	Ref.		Ref.	
Yes	-0.292 (-0.549, -0.034)	0.028	-0.013(-0.206, 0.233)	0.902
Electricity				
No	Ref.			
Yes	0.218 (-.020, 0.456)	0.071	0.056 (-0.145, 0.259)	0.566
ANC attendance				
No	Ref.			
Yes	0.237 (0.023, 0.451)	0.031	-0.005 (-0.155, 0.144)	0.942
Marital status of the parents				
Married	Ref.			
Cohabiting	0.117 (-0.154, 0.389)	0.381		
Not in union	-0.310 (-0.721, 0.101)	0.133		
Father’s education (years)	0.036 (0.001, 0.080)	0.113		
Current BF				
No	Ref.			
Yes	0.108 (-0.102, 0.319)	0.298		
Parity				
Primipara	Ref.			
Multipara	-0.048 (-0.385, 0.290)	0.773		
Water source				
Surface water or other	Ref.			
Borehole or tap	0.094 (-0.182, 0.370)	0.487		
Piped yard or home	0.235 (-0.343, 0.813)	0.409		

Ref.: comparison category. Regression modelling estimates are based on heteroscedasticity-robust standard errors.

## Discussion

This nested cohort study examined the effects of early childhood feeding on attained height among lower primary school-aged children in Mbale district followed up to 5–8 years age between 2006 and 2015. Baseline measures indicate that the prevalence of adequate complementary feeding was extremely low between 18–24 months of age with disparities by place of residence and parity of the mother. In fact, the prevalence of adequate feeding was so low using the strict IYCF definition that we used a modified definition to aid comparative assessment. Adequate CF at age 18–24 months appeared not to be associated with attained height whereas wealth status and place of residence were significantly associated with attained height between 5–8 years of age.

Only 2.32% of the children were receiving the adapted minimum acceptable diet between 18–24 months of age, based on the modified IYCF criteria. This percentage is below the 13% reported nationwide in the 2016 UDHS report [6] and figures from other developing countries. Recent findings from resource-constrained settings, including in Ethiopia [19, 20], Ghana [21] and India [22] have shown varying prevalence of minimum acceptable diet, yet consistently higher than that observed in this study. These disparities can be attributable at least partly to variability in operationalization of the IYCF criteria. It is also likely that poor knowledge about IYCF in this predominantly rural Ugandan population with a lower average education level compared to other studies could account for the inadequacy of child feeding in our study. It was suggested that Ugandan children born to mothers with lower literacy skills and without formal education were less likely to be fed adequately, given the role of literacy in information processing essential to ensure adequate child feeding and protection against undernutrition [23].

Our findings indicate a statistically insignificant effect of the type of complementary feeding on attained height in lower primary school-aged children. The statistical precision of this relatively negligible effect was sufficiently high, as reflected in narrow 95% confidence intervals. We deemed post-hoc power calculation less relevant given that it is increasingly seen as an obsolete and controversial practice, firmly criticized and discouraged [24]. This result is corroborated by those of sub-Saharan and South Asian studies, though in younger children [25–28]. However, a study in poor peri-urban and rural communities of Ecuador found a positive effect of complementary feeding on growth [29]. The absence of a statistically significant effect of complementary feeding on attained height in our study could be attributed to the lack of variability in child feeding since virtually all the children in this study were (quantitatively and qualitatively) receiving insufficient complementary feeding if one considers the strict IYCF criteria. Besides, these apparently opposing findings partly reflect the difference in designs of different studies: children were followed-up to 11 months in Ghana and 24 months in Ecuador against 5 to 8 years in our study. It is well established that growth velocity is intrinsically inconstant throughout childhood with a steadily fast linear growth during the first 2 years of life and a slow growth during the preschool years [30]. A study in healthy children followed up to 21 months has long revealed that growth is an episodic phenomenon alternating long stasis periods with short salutatory phases to the extent that there is virtually no growth during 90–95% of healthy infancy [31]. Additionally, only 3.61% of children in our study had meal frequency of three times and less than four in ten children were fed at least two times based on 24 dietary recall data, far below the minimum meal frequency of 3–4 times a day jointly recommended by WHO and UNICEF [32]. The likely low energy intake associated with inadequate meal frequency could have accounted for the absence of CF effect on child growth. It is being increasingly advocated that improvement in CF should focus on both energy density and feeding frequency and this can be achieved by scaling up properly tailored nutritional education in addition to provision of adequate complementary food [10, 11, 33].

We noted a strong negative linear correlation between child’s age at 5-8-years visit and HAZ. This association has also been consistently reported in studies conducted in other sub-Saharan countries [34, 35]. Two explanations are possible. First, there is a likely exacerbation of manifestations of prolonged nutritional deprivations with age. Second, as children continue growing up, they may get engaged in labor or schooling concurrently with suboptimal nutritional intake, and thus sink deeper in stunting [34, 36].

We found an improvement in attained HAZ between 18–24 months and 5–8 years of age with a substantial reduction in stunting rates independently of feeding status. This result is in the same direction as those from the Guatemalan study that showed better growth and recovery from malnutrition in children exposed to nutrition intervention in their first 3 years of life than those exposed in later childhood [37]. Our study highlights the necessity of investing in early childhood nutrition for short- and longer-term benefits. In addition, findings from this study support the perspective that the window of opportunity for catch-up growth may extend beyond the first 2 years of life as already remarked by others researchers [38–40].

One of the strengths of this study is the prospective nature of the nutritional data collection both in the first two years of life (18 to 24 months), a period now defined as the window of opportunity for nutritional interventions [41, 42], and in mid-childhood (5–8 years). To the best of our knowledge, this is the first study to prospectively examine the effect of early child feeding patterns, particularly complementary feeding on attained height among Ugandan children.

This study is limited by the low specificity of the definition used to operationalize child feeding. The strict IYCF definition could not be used in our analyses because there was no child who met all the criteria for minimum acceptable diet. Relaxing the IYCF criteria by generating a more sensitive definition of adequate child feeding might have introduced a potential for non-differential misclassification that could account for the apparent absence of effect of CF on child attained height. In addition, there is a potential for a conservative information bias that might have resulted from inaccurate dietary recall. Such a bias would have led to a non-differential misclassification of complementary feeding status (adequate or inadequate feeding). This was minimized by using a short dietary recall duration of 24 hours. In addition, the 7-day recall may not be fully representative of the entire period between 18–24 moths and 5–8 year periods. Accurate measurement of child feeding over time would be extremely resource intensive given its dynamic nature. The availability of food types changes with season and age related cultural practices and to adequately capture this is beyond the remit of the trial this study was nested in. This might have contributed to the non-differential misclassification of child feeding status.

Our analysis of complementary feeding did not focus on the frequency of consumption of specific food groups and items, but rather was focused on overall meal frequency and the consumption of specific food groups in line with WHO definitions [43]. Further research aiming at investigating the link between the frequency in which different food groups or items are consumed and child’s linear growth. There was a potential for a selection bias that might have occurred as a result of a possible selective loss to follow-up in children inadequately fed. However, a sensitivity analysis conducted using an inverse-probability weighting approach did not show any statistically significant change in the adjusted effect of adequate CF on attained height (β = -0.078; 95% CI: -0.339, 0.183; p = 0.560). Lastly, a residual confounding was likely since, given the availability of the data, a number of nutrition-sensitive factors could not be directly controlled for, including maternal mental health, health and family planning services, child protection, sanitation and hygiene. It can be argued that such factors could influence the scale, coverage, and effectiveness of nutrition-specific interventions, including complementary feeding [10, 44]. Improving nutrition alone is not enough and may not have the intended effects since many other factors impede growth of children [45].

## Conclusion

This predominantly rural population had a high prevalence of inadequate CF. Adequate CF between18-24 months of age did not have a strong effect on attained height in later childhood, though children adequately fed at 5–8 years of age tended to catch up with attained height better than those who were not. Other strategies need to be studied to improve child nutrition and linear growth in resource-limited settings.

## Supporting information

S1 Questionnaire PROMISE EBF questionnaire used for data collection at the 3-week visit. (DOC)

S2 Questionnaire PROMISE EBF questionnaire used for data collection at the 24-week visit. (DOC)

S3 Questionnaire PROMISE EBF questionnaire for the 18-month interview. (DOC)

S1 Dataset Minimal data set with variables used in the final multivariable regression analysis. The data set is in Stata 15 format and includes the variables cluster, residence, marital status, parity, wealth status, water source, exclusive breastfeeding, breastfeeding at age 18–24 months, antenatal care attendance, mother’s education, father’s education, electricity at home, adequate complementary feeding at 18–24 months of age, adequate child feeding at 5–8 years of age, height-for-age at age 18–24 months, height-for-age at 5–8 years and weight-for-height at age 18–24 months.
(DTA)

S1 Table Unadjusted and multivariable linear regression analyses of the association between the contextualized adequate CF at 18–24 months of age and attained height (HAZ) at age 5–8 years, without HAZ and WHZ at baseline. (DOCX)

## Abbreviations

CF: Complementary Feeding
DALYs: Disability Adjusted Life Years
DHS: Demographic and Health Survey
e.g.: for example (from Latin “exempli gratia”)
EBF: Exclusive Breast feeding
IQ: Intelligence Quotient
IQR: interquartile range
IYCF: Infant and Young Children Feeding
LAZ: Length-for-age z-score
bMCA: Multiple Correspondence Analysis
MICS: Multi Indicator Cluster Survey
MOH: Ministry Of Health
PR: Prevalence Rate
PROMISE EBF: Promoting infant health and nutrition in Sub-Saharan Africa: Safety and efficacy of exclusive breastfeeding
SD: Standard Deviation
SES: socio-economic status
TOVA: test of variables of attention
UBOS: Uganda Bureau of Statistics
UNICEF: United Nations International Child Emergency Fund
USA: United States of America
WAZ: Weight-for-age z-score
WHO: World Health Organisation
WLZ: Weight-for-length z-score
