# Supplementary material for: Effects of complementary feeding on attained height among lower primary school-aged children in Eastern Uganda: A nested prospective cohort study
Source: PLoS One. 2019 Feb 7;14(2):e0211411. doi: 10.1371/journal.pone.0211411 (PMC6366764; doi:10.1371/journal.pone.0211411)
Supplement: S1 Questionnaire — (DOC) [file pone.0211411.s001.doc]

# B: 3 week interview (Data Item Code (DIC): 03Q

**EH = Epi Handy
Radio Button (RB), One alternative only
Check Boxes (CB), Multiple alternatives allowed
Other alternatives: text (tx) and numeric (Num) (The rest: See EpiHandy manuals and separate entry SOP)**

**Lumasaaba = Local Language Uganda, will be corrected later in Uganda/Will have to be replaced with other local languages and French (Column 3. and 4. )**

**Column 5: Skip instructions EH/Paper + other comments on content**

**Coumn 6: Entry rule in EH + var.name (VN), Alternative coding (a)**

**Remember: EpiHandy has a special function for Do not know and Not applicable. It is only mentioned in the answer column when this is a natural/expectied answer for a question. We will be able to record DNK even if it is not written as an answer option.**

**Rule for all categorical answers: DNK tick in under question mark**

**Rule for all numerical answers: DNK = 99**

## SECTION 0 Introduction

EpiHandy p. a (1)

| **1. QUESTION ENGLISH** | | **2. ANSWER ENGLISH** | **3. QUESTION LUMASAABA** | | **4. ANSWER LUMASAABA** | **5. SKIP INSTRUCTION** | **6. COLUMN FOR CODING** |
| --- | --- | --- | --- | --- | --- | --- | --- |
| 1. Country/Site | 1. Burkina Faso 2. Uganda: MM 3. Uganda: B  4. Zambia: Site 1  5. Zambia: Site 2  6. SA Paarl 7. SA Rietveli 8. SA Umlazi | | |  |  | **NB! Necessary skip instructions should be given for currency and other country specific questions based in this question** | EH: RB VN: 03a01  a1=40 a2=51 a3=52 a4=61 a5=62 a6=71 a7=72 a8=73 |
| 2. Interviewer | _________ | | |  | 1. DONA 2. EVNA  3: FRWE 4. HEMU  5. MAKI  6. RANA  7. ZANG  8. Other, specify |  | EH: RB  a8: Tx  4 LETTER CODE;  UPPER CASE  Choose between drop down list/text  VN: 03a02  **VN:ALTERNATIVES:**  **KEEP 4 DIGIT CODE** |
| 3. Date: |  | | |  |  | Optional: Can be deleted as an entry alternative this is automatic in EH  I suggest we keep it in case the interview needs to be re-entered in time (audio/paper duplicate). | EH: Date/Mand  VN: 03a03 |
| 4. Time |  | | |  |  | Optional: Can be deleted as an entry alternative as it is automatic in EH  I suggest we keep it in case the interview needs to be re-entered in time (audio/paper duplicate). | EH: Time/Mand  VN: 03a04 |
| 5. GPS | 1. Long  2. Lat  3. Alt | | |  |  | Optional: Can be deleted as an entry alternative for those who will have this automatic in EH  Needs a cable in addition to the GPS  Alt recorded in m | EH: GPS VN: 03a05 (a1-a3)  a1:E/(W) ###°##.###  a2: N/S ##°##.###  a3: #### (#-####) |
| 6. Participant Id no/ Unique Subject Identifier (USI) | | #### | |  |  |  | EH: Num  VN: 03a06  4 digit code starting at 1001 all sites |

| 7. The mother has moved after the recruitment interview | 1. [_] Yes  2. [_] No |  |  | **SKIP:** If no skip to Initial Screen  Disable 8-13 in initial settings; if moved enable all | EH: RB  VN: 03a07 |
| --- | --- | --- | --- | --- | --- |
| 8. The mother has moved outside the cluster borders | 1. [_] Yes , specify where to  2. [_] No |  |  |  | EH: RB  VN: 03a08  a1: tx |
| 9. The mother has moved to another study cluster | 1. [_] Yes  2. [_] No |  |  |  | EH: RB  VN: 03a09 |

|  | **Do not read out:**  10. Note Sub-County/Division | **Do not read out:**  11. Note ward/parish=  CLUSTER CODE  (In Uganda) | 12. **Read English:** What is the name of your village/cell?  **Read Lugishu:** Lisina lye shishalo shoowo,  namwe khasiintsa khoowo bakhalanga barye? | **CODING!** |
| --- | --- | --- | --- | --- |
|  | 1. [_] Nakaloke  2. [_] Namanyonyi  3. [_] Bung.-Mutoto  4. [_] Bukonde  5. [_] Bunghoko  6. [_] Busoba  7. [_] Busiu  8. [_] Bukiende  9. [_] Industrial  10. [_] Nothern  11. [_] Wanale | 1. [_] Nakaloke  2. [_] Namunsi  3. [_] Kireka  4. [_] Namanyonyi  5. [_] Namagumba  6. [_] Bumuluya  7. [_] Bukasakya  8. [_] Bumboi  9. [_] Bumutoto  10. [_] Bubirabi  11. [_] Bukhumwa  12. [_] Bumbobi  13. [_] Bumasikye Busoba  14. [_] Bunamini  15. [_] Bufukhula  16. [_] Bunambutye  17. [_] Bumasikye Busiu  18. [_] Bunashimolo  19. [_] Namatala S  20. [_] Namatala D  21. [_] Malukhu  22. [_] Namakwekwe  23. [_] Nkoma  24. [_] Mohín | 1a. [_] Kolonyi I B  1b. [_] Kolonyi I C  2. [_] Namunsi cent B, part 1  3. [_] Kireka mile 6  4. [_] Namanyonyi central  5. [_] Namagumba  6. [_] Bumuluya Upper  7. [_] Munkaga B  8a. [_] Kamisyo  8b. [_] Nalwoka  9. [_] Bunamwani  10. [_] Makambo  11a. [_] Nambiti  11b. [_] Luyekhe  12a. [_] Bukumeka II A  12b. [_] Bukumeka II B  13. [_] Mahanga  14a. [_] Lwangoli  14b. [_] Buwangolo  15a. [_] Bumulahawasu  15b. [_] Bululsambu A  16a. [_] Musese Wapomokha  16b. [_] Musese Nakunuku  17a. [_] Wokukiri A  17a. [_] Wokukiri B  18a. [_] Bumahena  18b. [_] Nabikhoso  19. [_] Sisye cell B  20. [_] Doko cell C  21a. [_] Muti cell A  21b. [_] Muti cell B  21c. [_] Muti cell C  22a. [_] Mugisu cell A  22b. [_] Mugisu cell B  22c. [_] Kachumbala  23a. [_] Bujoroto cell B  23b. [_] Bujoroto cell D  24a.[_]Naksibisho cell A  24b.[_]Naksibisho cell B  24c. [_] Nagudi  25. [_] Other, specify | See recruitment interview! |

## INTIAL SCREENING QUESTONS ABOUT THE MOTHER - INFANT PAIR

**To be confirmed, not to be asked literally**

EpiHandy p b (2)

| **1. QUESTION ENGLISH** | **2. ANSWER ENGLISH** | **5. SKIP/RULES** | **6. COLUMN FOR CODING** |
| --- | --- | --- | --- |

| 1. She is the mother of the baby | 1. [_] Yes  2. [_] No | **SKIP: If no,**  **Discontunue from Section (S) I** | EH: RB  VN: 03b01 |
| --- | --- | --- | --- |
| 2. It was a single birth | 1. [_] Yes  2. [_] No | **SKIP: If no,**  **Discontunue from SI** | EH: RB  VN: 03b02 |
| 3. Severe malformation | 1. [_] Yes  2. [_] No | **SKIP: If yes,**  **Discontunue from SI** | EH: RB  VN: 03b03 |
| 4. The baby is dead | 1. [_] Yes  2. [_] No | **SKIP: If yes,**  **Discontunue from SI**  **ADMINISTER INFANT VERBAL AUTOPSY FORM**  **(SEPARATE DOCUMENT)** | EH: RB  VN: 03b04 |
| 5. The mother is dead | 1. [_] Yes  2. [_] No | **SKIP: If yes,**  **Discontunue from SI ADMINISTER MATERNAL VERBAL AUTOPSY FORM**  **(SEPARATE DOCUMENT)** | EH: RB  VN: 03b05 |
| 6. The mother is away for other reasons | 1. [_] Yes  2. [_] No | **SKIP: If yes,**  **Discontunue from SI**  **ADMINISER MISSED VISIT/LOSS/TERMINATION FORM (SEPARATE DOCUMENT)** | EH: RB  VN: 03b06 |
| 7. Planned revisit | **Date:** | **To be disabled and activated only if 1 is no and 7 is yes** | EH: Date  VN: 03b07 |

## SECTION I Questions about the baby:

**E**piHandy p. c (3)

| **1. QUESTION ENGLISH** | **2. ANSWER ENGLISH** | **5. SKIP/RULES** | **6. COLUMN FOR CODING** |
| --- | --- | --- | --- |
| 1. What is your baby’s birth date? | ___/____/_______  (dd/mm/yyyy) | **PROBE: If she does not know; get is as exact as possible** | EH: BIRTH DATE FORM  VN: 03c01 |
| 2. Do you have a Child Health Card or any other health card or book for your baby? | 1. [_] Yes  2. [_] No ↓ | **SKIP: If no, skip to q. 5 and skip q. 7**  ASK FOR: COUNTRY SPECIFIC HEALTH CARD | EH: RB  VN: 03c02U  Country specific health card |
| 3. May I please see it? | 1. [_] Yes  2. [_] No ↓ | **SKIP: If no, skip confirmation of BIRTH DATE and BIRTH WEIGHT q. 4 and 7 + VACCINATION SV q. 5**  **Keep the card nearby till the vaccination questions are finished** | EH: RB  VN: 03c03 |
| **Do not read out:**  4. Write down birth date written in the card: | Birth day written in the card:  ___/____/_______  (dd/mm/yyyy) |  | EH: Date  VN: 03c04 |
| 5. Was the child weighed at birth? | 1. [_] Yes  2. [_] No ↓ | **SKIP: If no, skip to q.8** | EH: RB  VN: 03c05 |
| 6. What was the birth weight? | 1. Mother’s answer:  _______kg (#.#)  [_] Do not remember |  | EH: RB  a1: Num (#.#)  a2: Nothing  VN: 03c06  a1: VN: 03c06a1 |
| **Do not read out:**  7. Birth weight written in the health card: | 1. Birth weight written in the card:  _______kg (#.#)  2. [_] Weight not indicated at the card |  | EH: Num  a1: Num (#.#)  a2: Nothing  VN: 03c07 |
| 8. What is the name of the child? | <NAME>  ______________ |  | EH: Tx  VN: name |
| 9. Is <NAME> a boy or a girl? | 1. [_] Girl  2. [_] Boy |  | EH: RB  VN: 03c09 |
| 10. Where did the birth take place? | 1. [_] At home  2. [_] At the traditional birth attendants place  3. [_] At the local maternity  4. [_] At the clinic  5. [_] At the hospital  6. [_] During transport to a health facility  7. [_]Other, specify |  | EH: RB  VN: 03c10 |
| 11. Who assisted you? | 1. [_] No one  2. [_] The traditional birth attendants  3. [_] A nurse/  midwife  4. [_] A doctor/clinical officer  5. [_] Any other health personell, not nurse or doctor  6. [_] Friends/ family  7. [_]Other, specify  _________________ |  | EH: CB  VN: 03c11 |
| 12. What kind of birth did you have?  Was it normal, c-section (caesarean) a breech or something else? | 1. [_] Normal vaginal  2. [_] Caesarean-section  3. [_] Breech  4. [_] Other, specify ________________ | Normal=Vaginal | EH: RB  VN: 03c12 |
| 13. Were there any problems during the birth? | 1. [_] Yes  2. [_] No ↓ | **SKIP: If no, skip to 15** | EH: RB  VN: 03c13 |
| 14. What kind of problem was that? | 1. [_] Needed technical assistance to get the baby out  2. [_] Had problems delivering the placenta  3. [_] Abnormal bleeding  4. [_] Needed abrupt caesarean section  5. [_] Other, specify  ________________ |  | EH: CB  a5: Tx  VN: 03c14 |
| 15. During your pregnancy, did you ever discuss with anyone at the antenatal clinic the best way for you to feed your baby? | 1. [_] Yes  2. [_] No |  | EH: RB  VN: 03c15 |

## SECTION II Initiation of breastfeeding:

EpiHandy p. d (4)

| **1. QUESTION ENGLISH** | **2. ANSWER ENGLISH** | **5. SKIP INSTRUCTION** | **6. COLUMN FOR CODING** |
| --- | --- | --- | --- |
| 1. Have you ever given breast milk to <NAME>? | 1. [_] Yes  2. [_] No ↓ | **SKIP: If no, skip q. SII 2,3**  **ALSO SKIP S III q. 1, 2, 3, 8,9,10, 12** | EH: RB  VN: 03d01 |
| 2. When did you put the baby to the breast after birth? | 1. [_] Within the first hour  2. [_] After the first hour and up to12 hours.  3. [_] After 12 hours and up to 24 hours  4. [_] After 24 hours and up to 48 hours (2^nd^ day)  5. [_] After 48 hours and up to 72 hours (3^rd^ day)  6. [_] After 72 hours (After the 3rd day) |  | EH: RB  VN: 03d02 |
| 3. Did you give the first milk to the baby or did you express and discard it? | 1. [_] Gave the first milk  2. [_] Expressed and discarded the first milk 3. [_] Both gave and expressed it  4. [_] Other, specify  ________________ |  | EH: RB  VN: 03d03 |
| 4. Within the first three days after birth, was <NAME> given anything to drink other than breast milk? (Many mouthfuls) | 1. [_] Yes  2. [_] No ↓ | **SKIP: If no, skip q. 6**  **Drink refers to the attempt of giving the newborn a meal** | EH: RB  VN: 03d04 |
| 5. Within the first days did the baby get anything to taste; a few drops of something or less than a mouth full? | 1. [_] Yes  2. [_] No ↓ | **SKIP: If no, skip q. 7**  **Taste refers to the practice of letting the baby taste a few drops or give a teaspoon with a liquid, often done because of traditions and rituals** | EH: RB  VN: 03d05 |
| 6. What was the child given to drink whitin the first 3 days after birth? **R** | 1. [_] Water  2. [_] Water with sugar (or glucose)  3. [_] Water with salt  4. [_] Diluted cow’s milk  5. [_] Not diluted cow’s milk  6. [_] Infant formula  7. [_] Any other powdered milk  8. [_] Any porridge  9. [_] Any soup  Type:____________  10. [_] Any liquid as part of a ritual.  11. [_] Alchohol  12. [_] Traditional medicine  13. [_] Non-prescribed medicine, specify ___________  14. [_] Prescribed medicine specify, __________________  15. [_] Honey  16. [_] Other, specify _________________ | **RULE:**  **Tick off all that apply**  **ASK for item by item**  **Any water with salt qualifies for alternative no 3** | EH: List w Yes/No buttons  a9: Tx  a13: Tx  a14: Tx  a16: Tx  VN: 03d06 |
| 7. What was the child given to taste | 1. [_] Water  2. [_] Water with sugar (or glucose)  3. [_] Water with salt  4. [_] Diluted cow’s milk  5. [_] Not diluted cow’s milk  6. [_] Infant formula  7. [_] Any other powdered milk  8. [_] Any porridge  9. [_] Any soup  Type:____________  10. [_] Any liquid as part of a ritual.  11. [_] Alchohol  12. [_] Traditional medicine  13. [_] Non-prescribed medicine, specify ___________  14. [_] Prescribed medicine specify, __________________  15. [_] Honey  16. [_] Other, specify _________________ |  | EH: List w Yes/No buttons  a9: Tx  a13: Tx  a14: Tx  a16: Tx  VN: 03d07 |

## SECTION III Infant feeding recalls and questions on mother’s health

**EpiHandy p. (e) 5**

| **1. QUESTION ENGLISH** | **2. ANSWER ENGLISH** | **5. SKIP INSTRUCTION** | **6. COLUMN FOR CODING** |
| --- | --- | --- | --- |
| 1. Do you still breastfeed <NAME>? | 1. [_] Yes ↓  2. [_] No | **SKIP: If yes, skip to q. 5** | EH: RB  VN: 03e01 |
| 2. Did you ever breastfed your child? | 1. [_] Yes *!5  2. [_] No ↓ | **SKIP: If no, skip to q. 4** | EH: RB  VN: 03e02 |
| 3. For how long did you breastfeed your child? | 1. Weeks: _______  2. [_] Do not know | **< 1 week = 0**  **Report in full weeks** | EH: RB  a1: Num  a2: Nothing  VN: 03e03 |
| 4. What were your reasons for stopping to breastfeed/not breastfeed your child? | 1. [_] Work  2. [_] Education  3. [_] Illness, other than lactation problems  4. [_] Lactation problems  5. [_] Child not grow well  6. [_] Child crying a lot  7. [_] Not enough breastmilk  8. [_] No answer  9. [_] Advice/pressure from others  10. [_] Other, specify ________________ |  | EH: CB  a9: Tx  VN: 03e04 |
| 5. Have you ever had any problem with your breast since your child was born? | 1. [_] Yes  2. [_] No ↓ | **SKIP: If no, skip to the dietary 24-hour recall and q. 8** | EH: RB  VN: 03e05 |
| 6. What did you have? | 1. [_] Engorgement  2. [_] Cracked nipples  3. [_] Abcess  4. [_] Infection  5. [_] Operation  6. [_] Trauma  7. [_] Other, specify  _______________ |  | EH: CB  VN: 03e06 |
| 7. How old was your baby when this occurred? | Weeks _______ | **< 1 week = 0**  **Report in full weeks** | EH: Num (#)  VN: 03e07 |

**Dietary 24-hour recall:**

**English:**

I am now going to ask you questions about what you fed your baby from the time you woke up yesterday morning till you woke up this morning.

**Lumasaaba:**

Ari itsya khukhureba bireebo biambagana khubyeesi walisiile umwaana woowo khukwaama wenyukhile ingolobe kumutikhini khukhwoleesa shalee lo kumutikhini

| **1. QUESTION ENGLISH** | **2. ANSWER ENGLISH** | **5. SKIP INSTRUCTION** | **6. COLUMN FOR CODING** |
| --- | --- | --- | --- |
| 8. From the time you woke up yesterday morning till you woke up this morning did you breastfeed your baby? | 1. [_] Yes  2. [_] No ↓ | **SKIP: If no, skip to q. 11** | EH: Tx  VN: 03e08 |
| 9. From the time you woke up yesterday morning till you went to bed last night, how many times did you breastfeed? | ______(#(#)) |  | EH: Num  VN: 03e09 |
| 10. From the time you went to bed last night till you woke up this morning, how many times did you breastfeed? | _______(#(#)) |  | EH: Num  VN: 03e10 |

| 11. From the time you woke up yesterday morning till you woke up this morning:  Did you give any of the following items to the child? And if you did,  will you please tell how many times you gave it? Did you give any:  1. Water Yes □ No □ Freq.[__]  2. Water with sugar or glucose Yes □ No □ Freq.[__]  3. Fruit juice Yes □ No □ Freq.[__]  4. Herbs Yes □ No □ Freq.[__]  5. Tea without milk Yes □ No □ Freq.[__]  6. Tea with milk Yes □ No □ Freq.[__]  7. Rice water Yes □ No □ Freq.[__]  8. Diluted cow’s milk Yes □ No □ Freq.[__]  9. Not diluted cow’s milk Yes □ No □ Freq.[__]  10. Infant formula Yes □ No □ Freq.[__]  11. Other powdered milk Yes □ No □ Freq.[__]  12. Dairy product like yoghurt, cream, sour milk  Yes □ No □ Freq.[__]  13. Goat’s milk Yes □ No □ Freq.[__]  14. Cereals, porridge,bread, fermented porridge  Yes □ No □ Freq.[__]  15. Fruits/vegetables Yes □ No □ Freq.[__]  16. Meat Yes □ No □ Freq.[__]  17. Fish Yes □ No □ Freq.[__]  18. Eggs Yes □ No □ Freq.[__]  19. Gripe water Yes □ No □ Freq. [__]  20. Non-prescribed medicine, specify  Yes □ No □ specify  21. Prescribed medicine, specify Yes □ No □ Specify  22. Alcohol like beer or brew Yes □ No □ Freq.[__]  23. Other, Specify Yes □ No □ specify  ____________________________________ | EH: List with Yes/No buttons  **NB: let all the specification options be Num! (99= DNK)**  VN: 03e11 |
| --- | --- |

**Dietary** 1 Week recall

| **1. QUESTION ENGLISH** | **2. ANSWER ENGLISH** | **5. SKIP INSTRUCTION** | **6. COLUMN FOR CODING** |
| --- | --- | --- | --- |
| 12. Thinking one week back, have you breastfed your baby? | 1. [_] Yes  2. [_] No |  | EH: RB  VN: 03e12 |

**Dietary Recall since birth**

| 13. Now I am going to ask you if you ever have given the following to your baby and if you have done that, please tell us when you did that for the first time:  1 Water Yes □ No □ Wk __  2. Water with sugar or glucose Yes □ No □ Wk __  3. Fruit juice Yes □ No □ Wk __  4. Herbs Yes □ No □ Wk __  5. Tea without milk Yes □ No □ Wk __  6. Tea with milk Yes □ No □ Wk __  7. Rice water Yes □ No □ Wk __  8. Diluted cow’s milk Yes □ No □ Wk __  9. Not diluted cow’s milk Yes □ No □ Wk __  10. Infant formula Yes □ No □ Wk __  11. Other powdered milk Yes □ No □ Wk __  12. Dairy product like yoghurt, cream, sour milk  Yes □ No □ Wk __  13. Goat’s milk Yes □ No □ Wk __  14. Cereals, porridge, bread, fermented porridge  Yes □ No □ Wk __  15. Fruits/vegetables Yes □ No □ Wk __  16. Meat Yes □ No □ Wk __  17. Fish Yes □ No □ Wk __  18. Eggs Yes □ No □ Wk __  19. Gripe water Yes □ No □ Wk __  20. Non-prescribed medicine, specify Yes □ No □ Type: 1^st^ time:  21. Prescribed medicine, specify Yes □ No □ Type: 1^st^ time:  22. Alcohol like beer or brew Yes □ No □  23. Other, Specify Yes □ No □ Type: 1^st^ time: | EH: List with Yes/No buttons  **NB: let all the specification options beNum! (DNK=99)** |
| --- | --- |

## SECTION IV Questions about leaving the child

**EpiHandy p. f (6)**

| **1. QUESTION ENGLISH** | **2. ANSWER ENGLISH** | **5. SKIP INSTRUCTION** | **6. COLUMN FOR CODING** |
| --- | --- | --- | --- |
| 1. Have you ever been separated from your child since child birth so that someone else has fed the child?/ Have you ever left your child since childbirth so that someone else has fed the child? | 1. [_] Yes  2. [_] No ↓ | **SKIP: If no, skip to S V**  **(Sentence 2 preferred by DC in Uganda; sentence no.1 is offending here)** | EH: RB  VN: 03f01 |
| 2. What did the one taking care of your child feed last time? | 1. [_] Water based liquids  2. [_] Milk based liquids/semi-solid feeds  3. [_] Expressed breast milk from the mother  4. [_] Expressed breast milk from another woman, not the mother  5. [_] Do not know  6. [_] Other, specify ________ | **RULE: Tick off all that apply**  **Do not ask from the list, but probe from it.** | EH: CB  a6: Tx  VN: 03f02 |
| 3. How often did it happen the last week that you had someone else to feed the child? | ____ Times/last week |  | EH: Num  VN: 03f03 |
| 4. How many times do you usually leave your baby per week? | ____ Times/week |  | EH: RB  VN: 03f04 |

## SECTION V Bed Net, vaccination and micronutrients

**EpiHandy p. g (7)**

Now I am going to ask you questions which are related to your baby’s health:

| **1. QUESTION ENGLISH** | **2. ANSWER ENGLISH** | **5. SKIP INSTRUCTION** | **6. COLUMN FOR CODING** |
| --- | --- | --- | --- |
| 1. Does <NAME> sleep in your bed? | 1. [_] Yes  2. [_] No |  | EH: RB  VN: 03g01 |
| 2. Is the baby covered by a bednet at night? | 1. [_] Yes  2. [_] No | **Both a separate net for the baby and a shared net with the mother qualifies for yes here** | EH: RB  VN: 03g02 |
| 3. Has <NAME> had any vaccinations? | 1. [_] Yes  2. [_] No ↓  3. [_] Do not know | **SKIP: If no, skip to q. 6**  **Train DC to probe for both injections and mouth drops** | EH: RB  VN: 03g03 |
| 4. Has your baby got the BCG vaccine? (mother’s answer) | 1. [_] Yes  2. [_] No  3. [_] Do not know | **Given right upper arm**  **(country specific)** | EH: RB  VN: 03g04U |
| 5. Has <NAME> got the polio vaccine? (The first one) (mother’s answer) | 1. [_] Yes  2. [_] No  3. [_] Do not know | **Given as mouth drops** | EH: RB  VN: 03g05U |
| Do not ask out loudly, but ask again to look at <NAME>’s card and:  6. Note down vaccinations given which is stated in the child health card. | 1. [_] BCG  2. [_] Polio O | **(P.S. In the Ugandan CHC it is written HebB and not HepB, tell this to the Ugandan DC)** | EH: List with Yes/No buttons  **JAMES/THORKILD: Optional:**  **Specify with dates!**  **SA drops this function. The Danes suggests it!**  VN: 03g06U |
| 20. Now I would like to ask you about yourself. Since you gave birth: Have you taken any of these Vit A supplements? | 1. [_] Yes  2. [_] No  3. [_] Do not know | **Rule: DC show the different types of vitamin A capsules that commonly are used** | EH: RB  VN:03g20 |
| 21. When you were pregnant did you take any of these iron tablets? | 1. [_] Yes, she identified that she had taken one or several of the iron tablets  2. [_] No, she confirmed that she had not taken any of the iron tablets  3. [_] She was not sure whether she had taken any of these iron tablets | **Show the different types of iron tablets that pregnant women commonly take**  **SKIP: If alternative 2, skip question 8 and ask question 9** | EH: RB  VN: 03g21 |
| 22. How many iron tablets did you take during the whole pregnancy? | 1. [_] 1-10  2. [_] 11-30  3. [_] More than 30  4. [_] Do not remember |  | EH: RB  VN: 03g22 |
| 23. Did you take any other tablets containing iron during your pregnancy? If so can you please show them to me? | 1. [_] No, did not take any other iron tablets.  2. [_] Yes, and she showed tablets that contains iron.  3. [_] Said yes and showed tablets with unclear content or without iron.  4. [_] Said yes, but did not have any tablets to show. | **Data collectors need to have a set of the most common iron tablets available so they can compare with those the woman show** | EH: RB  VN: 03g23 |

## VI Morbidity, VI A Diarrhoea

**Epihandy p. h (8)**

## Diarrhoea 24-hour recall

| **1. QUESTION ENGLISH** | **2. ANSWER ENGLISH** | **5. SKIP INSTRUCTION** | **6. COLUMN FOR CODING** |
| --- | --- | --- | --- |
| 1. From yesterday morning till this morning, did <NAME> have diarrhoea? | 1. [_] Yes  2. [_] No ↓ | **SKIP: If no, skip to Diarrhoea 2 week recall**  **DC: Diarrhoe = loose or watery stools (1 to n times)** | EH: RB  VN:03h01 |
| 2. Did <NAME> pass any watery stools? | 1. [_] Yes  2. [_] No | Watery stools= stools with no formed matter whatsoever | EH: RB  VN: 03h02 |
| 3. How many loose or watery stools did <NAME> pass? **R** | _______ (#(#)) |  | EH: Num  VN: 03h03 |
| 4. Did any of the stools contain blood? | 1. [_] Yes  2. [_] No |  | EH: RB  VN: 03h04 |
| 5. Were the stools of different consistency than before <NAME> fell ill with diarrhoea? | 1. [_] Yes  2. [_] No |  | EH: RB  VN: 03h05 |
| 6. Did the illness interfere with <NAME>’s ability to drink or eat? | 1. [_] Yes  2. [_] No |  | EH: RB  VN: 03h06 |
| 7. Did you seek treatment for <Name>? | 1. [_] Yes  2. [_] No ↓ | **SKIP: If no, skip to q. 9** | EH: RB  VN: 03h07 |
| 8. Where did you go? | 1. [_] Relatives and friends  2. [_] Traditional healer  3. [_] Drugshop/ Pharmacy  4. [_] Government or private clinic/ surgery/community health centre including general practitioner  5. [_] The emergency/ outpatient department of a hospital  6. [_] Other, specify ________________ |  | EH: CB  a6: Tx  VN:  03h08 |
| 9. Was the child admitted to a hospital? | 1. [_] Yes  2. [_] No ↓ | **SKIP: If no, skip to Diarrhoea 2 week recall** | EH: RB  VN: 03h09 |
| 10. Please give name of hospital? | 1. [_] Mbale Main hospital  2. [_] Bududa Hospital  3. [_] Busiu  4. [_] Bushacori  5. [_] SIRA  6. [_] Bufumbo  7. [_] Mission  8. [_] JOY  9. [_] Cure Hospital  10. [_] Ahamedia  11. [_] St. Martin  12. [_] Other, specify ________________ |  | EH: RB  VN: 03h10  **VN UGANDA:**  **03h10U** |
| 11. Was this the nearest health unit? | 1. [_] Yes  2. [_] No ↓ | **SKIP: If yes, skip to Diarrhoea 2 week recall** | EH: RB  VN: 03h11 |
| 12. Why did you go there? | 1. [_] Health cervices better than at the nearest health unit  2. [_] Transport was available  3. [_] The nearest health unit is more expensive than the one I went to  4. [_] I wanted to go to the biggest hospital I can afford  5. [_] I do not trust the people at the nearest health unit  6. [_] Other, specify |  | EH: CB  a6: Tx  VN:  03h12 |

## Diarrhoea 2 week recall

Epihandy p. i (9)

| **1. QUESTION ENGLISH** | **2. ANSWER ENGLISH** | **5. SKIP INSTRUCTION** | **6. COLUMN FOR CODING** |
| --- | --- | --- | --- |
| 1. During the last two weeks that ended yesterday morning, did <NAME> have diarrhoea? | 1. [_] Yes  2. [_] No ↓ | **SKIP: If no, skip to 24-hour recall for pneumonia/ALRI questions**  **DC: Diarrhoea = loose or watery stools (1 to n times)** | EH: RB  VN: 03i01 |
| 2. Did <NAME> pass any watery stools? | 1. [_] Yes  2. [_] No | Watery stools= stools with no formed matter whatsoever | EH: RB  VN: 03i02 |
| 3. The day <NAME> had most loose or watery stools, how many loose or watery stools did <NAME> pass? **R** | _______ (#(#)) | **RULE: Write 99 for number of stools if informant does not remember** | EH: Num  VN: 03i03 |
| 4. Did any of the stools contain blood? | 1. [_] Yes  2. [_] No |  | EH: RB  VN: 03i04 |
| 5. Were the stools of different consistency than before <NAME> fell ill with diarrhoea? | 1. [_] Yes  2. [_] No |  | EH: RB  VN: 03i05 |
| 6. Did the illness interfere with <NAME>’s ability to drink or eat? | 1. [_] Yes  2. [_] No |  | EH: RB  VN: 03i06 |
| 7. Did you seek treatment for <Name>? | 1. [_] Yes  2. [_] No ↓ |  | EH: RB  VN: 03i07 |
| 8. Where did you go? | 1. [_] Relatives and friends  2. [_] Traditional healer  3. [_] Drugshop/ Pharmacy  4. [_] Government or private clinic/ surgery/community health centre including general practitioner  5. [_] The emergency/ outpatient department of a hospital  6. [_] Other, specify ________________ |  | EH: CB  a6: Tx  VN:  03i08 |
| 09. Was the child admitted to a hospital? | 1. [_] Yes  2. [_] No ↓ | **SKIP: If no, skip to q.13** | EH: RB  VN: 03i09 |
| 10. Please give name of hospital? | 1. [_] Mbale Main hospital  2. [_] Bududa Hospital  3. [_] Busiu  4. [_] Bushacori  5. [_] SIRA  6. [_] Bufumbo  7. [_] Mission  8. [_] JOY  9. [_] Cure Hospital  10. [_] Ahamedia  11. [_] St. Martin  12. [_] Other, specify ________________ |  | EH: RB  VN: 03i10  **VN UGANDA:**  **03i10U** |
| 11. Was this the nearest health unit? | 1. [_] Yes  2. [_] No ↓ | **SKIP: If no, skip to q. 16** | EH: RB  VN: 03i11 |
| 12. Why did you go there? | 1. [_] Health cervices better than at the nearest health unit  2. [_] Transport was available  3. [_] The nearest health unit is more expensive than the one I went to  4. [_] I wanted to go to the biggest hospital I can afford  5. [_] I do not trust the people at the nearest health unit  6. [_] Other, specify |  | EH: CB  a6: Tx  VN:  03i12 |
| 13. ALT 1: How many days did the diarrhoea last? |  | **(Only to be enabeled if no at the 24 hour recall and yes in the 2 week recall)** | EH: Num  VN: 03i13 |
| 14. ALT 2: How many days has the diarrhoea lasted? |  | **(Only to be anabeled if yes at the 24-hour recall and yes in the 2 week recall)** | EH: Num  VN: 03i14 |
| 15. During this period of illness you have described, did you change the way you were feeding your child in any way? |  | **SKIP: If no, skip to pneumonia q. 17** | EH: RB  VN: 03i15 |
| 16. In which way? | 1. [_] Stopped breast feeding  2. [_] Stopped non-human milk  3. [_] Stopped other liquids  4. [_] Stopped solid foods  5. [_] Only breast fed at night  6. [_] Began giving other liquids  7. [_] Began giving solid foods  8. [_] Other, specify |  | EH: CB  a8: Tx  VN: 03i16 |
| 17. During the period of illness did you feed your baby more often, more seldom than or just as often as before the illeness started? | 1. [_] More often  2. [_] More seldom than before the illness started  3. [_] Did not change feeding frequency. |  | EH: RB  VN: 03i17 |

## VI B ALRI/pneumonia: Pneumonia 24-hour recall

## Pneumonia 24-hour recall

EpiHandy p. j (10)

| **1. QUESTION ENGLISH** | **2. ANSWER ENGLISH** | **5. SKIP INSTRUCTION** | **6. COLUMN FOR CODING** |
| --- | --- | --- | --- |
| 1. From yesterday morning till this morning, did <NAME> have cough? | 1. [_] Yes  2. [_] No |  | EH: RB  VN: 03j01 |
| 2. From yesterday morning till this morning, did <NAME> have fast or difficult breathing? | 1. [_] Yes  2. [_] No ↓ | **SKIP: If no q. 1 and 2 skip to Pneumonia 2 week recall** | EH: RB  VN: 03j02 |
| 3. Did the illness interfere with <NAME>’s ability to drink or eat? | 1. [_] Yes  2. [_] No |  | EH: RB  VN: 03j03 |
| 4. Was <NAME> admitted to a hospital for the illness? | 1. [_] Yes  2. [_] No ↓ | **SKIP: If no, skip to Pneumonia 2 week recall** | EH: RB  VN: 03j04 |
| 5. Please give name of hospital? | 1. [_] Mbale Main hospital  2. [_] Bududa Hospital  3. [_] Busiu  4. [_] Bushacori  5. [_] SIRA  6. [_] Bufumbo  7. [_] Mission  8. [_] JOY  9. [_] Cure Hospital  10. [_] Ahamedia  11. [_] St. Martin  12. [_] Other, specify ________________ |  | EH: RB  VM: 03j05  **VN UGANDA:**  **03j05U** |
| 6. Was this the nearest health unit? | 1. [_] Yes  2. [_] No ↓ | **SKIP: If yes, skip to Pneumonia 2 week recall** | EH: RB  VN: 03j06 |
| 7. Why did you go there? | 1. [_] Health cervices better than at the nearest health unit  2. [_] Transport was available  3. [_] The nearest health unit is more expensive than the one I went to  4. [_] I wanted to go to the biggest hospital I can afford  5. [_] I do not trust the people at the nearest health unit  6. [_] Other, specify |  | EH: CB  a6: Tx  VN:  03j07 |

## Pneumonia 2 Week recall

EpiHandy p. k (11)

| **1. QUESTION ENGLISH** | **2. ANSWER ENGLISH** | **5. SKIP INSTRUCTION** | **6. COLUMN FOR CODING** |
| --- | --- | --- | --- |
| 1. During the last two weeks that ended yesterday morning, did <NAME> have cough? | 1. [_] Yes  2. [_] No |  | EH: RB  VN: 03k01 |
| 2. During the last two weeks that ended yesterday morning, did <NAME> have fast or difficult breathing? | 1. [_] Yes  2. [_] No ↓ | **SKIP: If no q. 1 and 2 skip to hospitalization section** | EH: RB  VN: 03k02 |
| 3. Did the illness interfere with <NAME>’s ability to drink or eat? | 1. [_] Yes  2. [_] No |  | EH: RB  VN: 03k03 |
| 4. Was <NAME> admitted to a hospital for the illness? | 1. [_] Yes  2. [_] No ↓ | **SKIP: If no, skip to q. 8** | EH: RB  VN: 03k04 |
| 5. Please give name of hospital? | 1. [_] Mbale Main hospital  2. [_] Bududa Hospital  3. [_] Busiu  4. [_] Bushacori  5. [_] SIRA  6. [_] Bufumbo  7. [_] Mission  8. [_] JOY  9. [_] Cure Hospital  10. [_] Ahamedia  11. [_] St. Martin  12. [_] Other, specify ________________ |  | EH: RB  VN: 03k05  **VN UGANDA:**  **03k05U** |
| 6. Was this the nearest health unit? | 1. [_] Yes  2. [_] No ↓ | **SKIP: If no, skip to q. 8** | EH: RB  VN: 03k06 |
| 7. Why did you go there? | 1. [_] Health cervices better than at the nearest health unit  2. [_] Transport was available  3. [_] The nearest health unit is more expensive than the one I went to  4. [_] I wanted to go to the biggest hospital I can afford  5. [_] I do not trust the people at the nearest health unit  6. [_] Other, specify |  | EH: CB  a6: Tx  VN:  03k07 |
| 8. During this period of illness you have described, did you change the way you were feeding your child in any way? | 1. [_] Yes  2. [_] No ↓ | **SKIP: If yes, skip to q. 10** | EH: RB  VN: 03k8 |
| 9. In which way? | 1. [_] Stopped breast feeding  2. [_] Stopped non-human milk  3. [_] Stopped other liquids  4. [_] Stopped solid foods  5. [_] Only breast fed at night  6. [_] Began giving other liquids  7. [_] Began giving solid foods  8. [_] Other, specify |  | EH: CB  a8: Tx  VN: 03k9 |
| 10. During the period of illness did you feed your baby more often, more seldom than or just as often as before the illeness started? | 1. [_] More often  2. [_] More seldom than before the illness started  3. [_] Did not change feeding frequency. |  | EH: RB  VN: 03k10 |

## VI C Hospitalizations

**EpiHandy p. l (12)**

| **1. QUESTION ENGLISH** | **2. ANSWER ENGLISH** | **5. SKIP INSTRUCTION** | **6. COLUMN FOR CODING** |
| --- | --- | --- | --- |
| 1. Since birth has <NAME> ever been admitted to hospital? | 1. [_] Yes  2. [_] No ↓ | **SKIP: If no, skip to S VII** | EH: RB  VN: 03l01 |
| 2. How many times has <NAME> been admitted to hospital? | _____ (#(#)) |  | EH: Num  VN: 03l02 |
| 3. How old in weeks was your baby (each time) when he/she was in hospital? | 1. [_] 1^st^ time ______  2. [_] 2^nd^ time ______  3. [_] 3rd time _____  4. [_] 4th time ______  5. [_] 5^th^ time ______  6. [_] 6^th^ time ______  7. [_] 7^th^ time ______  8. [_] 8^th^ time ______ | **RULE: Tick off all that apply** | EH: CB  a1: Num  a2: Num  a3: Num.  VN: 03l03  . |
| 4. For how many days was <NAME> (each time) in hospital? | 1. [_] 1^st^ time ______  2. [_] 2^nd^ time ______  3. [_] 3rd time _____  4. [_] 4th time ______  5. [_] 5^th^ time ______  6. [_] 6^th^ time ______  7. [_] 7^th^ time ______  8. [_] 8^th^ time ______ |  | EH: CB  a1: Num  a2: Num  a3: Num etc.  VN: 03l04 |
| 5. What was the reason <NAME> was in the hospital each time: | 1. [_] 1^st^ time ______  2. [_] 2^nd^ time ______  3. [_] 3rd time _____  4. [_] 4th time ______  5. [_] 5^th^ time ______  6. [_] 6^th^ time ______  7. [_] 7^th^ time ______  8. [_] 8^th^ time ______ | **RULE: ENTER THE CORRECT NUMBER FROM THE LIST BELOW**  1 = Diarrhoea  2 = Pneumonia/ “Cough and difficult breathing”  3 = Malaria  4 = Accident  5 = specify what ___________ | EH: CB  a1: Tx  a2: Tx  a3: Tx etc.  VN: 03l05 |

## VII Anthropometry

**EpiHandy p. m (13)**

| 1. Baby’s weight | _____________kg (#.#) |  |  | EH: Num  VN:03m01 |
| --- | --- | --- | --- | --- |
| 2. Baby’s length ______ | _____________cm (##.#) |  |  | EH: Num  VN: 03m02 |
| 3: Other comments |  |  |  | EH: Large text field  VN: 03m03 |
