# Supplementary material for: Effects of complementary feeding on attained height among lower primary school-aged children in Eastern Uganda: A nested prospective cohort study
Source: PLoS One. 2019 Feb 7;14(2):e0211411. doi: 10.1371/journal.pone.0211411 (PMC6366764; doi:10.1371/journal.pone.0211411)
Supplement: S3 Questionnaire — (DOC) [file pone.0211411.s003.doc]

# B: 18 month interview (Data Item Code (DIC): 24Q

**EH = Epi Handy
Radio Button (RB), One alternative only
Check Boxes (CB), Multiple alternatives allowed
Other alternatives: text (tx) and numeric (Num) (The rest: See EpiHandy manuals and separate entry SOP)**

**Lumasaaba = Local Language Uganda, will be corrected later in Uganda/Will have to be replaced with other local languages and French (Column 3. and 4. )**

**Column 5: Skip instructions EH/Paper + other comments on content**

**Coumn 6: Entry rule in EH + var.name (VN), Alternative coding (a)**

**Remember: EpiHandy has a special function for Do not know and Not applicable. It is only mentioned in the answer column when this is a natural/expectied answer for a question. We will be able to record DNK even if it is not written as an answer option.**

**Rule for all categorical answers: DNK tick in under question mark**

**Rule for all numerical answers: DNK = 99**

## SECTION 0 Introduction

EpiHandy p. a (1)

| **1. QUESTION ENGLISH** | | **2. ANSWER ENGLISH** | **3. SKIP INSTRUCTION** | **4. COLUMN FOR CODING** |
| --- | --- | --- | --- | --- |
| 1. Country/Site | 1. Burkina Faso 2. Uganda: MM 3. Uganda: B  4. Zambia: Site 1  5. Zambia: Site 2  6. SA Paarl 7. SA Rietveli 8. SA Umlazi | | **NB! Necessary skip instructions should be given for currency and other country specific questions based in this question** | EH: RB/Mand VN: 1Yra01  a1=40 a2=51 a3=52 a4=61 a5=62 a6=71 a7=72 a8=73 |
| 2. Interviewer | 1. DONA 2. EVNA  3: FRWE 4. HEMU  5. MAKI  6. RANA  7. ZANG  8. Other, specify | |  | EH: RB/Mand  a8: Tx  4 LETTER CODE;  UPPER CASE  Choose between drop down list/text  VN: 1Yra02  **VN:ALTERNATIVES:**  **KEEP 4 DIGIT CODE** |
| 3. Date: |  | | Optional: Can be deleted as an entry alternative this is automatic in EH  I suggest we keep it in case the interview needs to be re-entered in time (audio/paper duplicate). | EH: Date/Mand  VN: 1Yra03 |
| 4. Time |  | | Optional: Can be deleted as an entry alternative as it is automatic in EH  I suggest we keep it in case the interview needs to be re-entered in time (audio/paper duplicate). | EH: Time/Mand  VN: 1Yra04 |
| 5. GPS | 1. Long  2. Lat  3. Alt | | Optional: Can be deleted as an entry alternative for those who will have this automatic in EH  Needs a cable in addition to the GPS  Alt recorded in m | EH: GPS VN: 1Yra05 (a1-a3)  a1:E/(W) ###°##.###  a2: N/S ##°##.###  a3: #### (#-####) |
| 6. Participant Id no/ Unique Subject Identifier (USI) | | #### |  | EH: Num  VN: 1Yra06  4 digit code starting at 1001 |

| 7. The mother has moved after the 24 week interview | 1. [_] Yes  2. [_] No | **SKIP:** If no skip to  Initial screen | EH: RB  VN: 1Yra07 |
| --- | --- | --- | --- |
| 8. The mother has moved outside the cluster borders | 1. [_] Yes  2. [_] No |  | EH: RB  VN: 1Yra08 |
| 9. The mother has moved to another study cluster | 1. [_] Yes  2. [_] No |  | EH: RB  VN: 1Yra09 |

|  | **Do not read out:**  10. Note Sub-County/Division | **Do not read out:**  11. Note ward/parish=  CLUSTER CODE  (In Uganda) | 12. **Read English:** What is the name of your village/cell?  **Read Lugishu:** Lisina lye shishalo shoowo,  namwe khasiintsa khoowo bakhalanga barye? | **CODING!** |
| --- | --- | --- | --- | --- |
|  | 1. [_] Nakaloke  2. [_] Namanyonyi  3. [_] Bung.-Mutoto  4. [_] Bukonde  5. [_] Bunghoko  6. [_] Busoba  7. [_] Busiu  8. [_] Bukiende  9. [_] Industrial  10. [_] Nothern  11. [_] Wanale  12. [_] Other, specify | 1. [_] Nakaloke  2. [_] Namunsi  3. [_] Kireka  4. [_] Namanyonyi  5. [_] Namagumba  6. [_] Bumuluya  7. [_] Bukasakya  8. [_] Bumboi  9. [_] Bumutoto  10. [_] Bubirabi  11. [_] Bukhumwa  12. [_] Bumbobi  13. [_] Bumasikye Busoba  14. [_] Bunamini  15. [_] Bufukhula  16. [_] Bunambutye  17. [_] Bumasikye Busiu  18. [_] Bunashimolo  19. [_] Namatala S  20. [_] Namatala D  21. [_] Malukhu  22. [_] Namakwekwe  23. [_] Nkoma  24. [_] Moni  25. [_] Other, specify | 1a. [_] Kolonyi I B  1b. [_] Kolonyi I C  2. [_] Namunsi cent B, part 1  3. [_] Kireka mile 6  4. [_] Namanyonyi central  5. [_] Namagumba  6. [_] Bumuluya Upper  7. [_] Munkaga B  8a. [_] Kamisyo  8b. [_] Nalwoka  9. [_] Bunamwani  10. [_] Makambo  11a. [_] Nambiti  11b. [_] Luyekhe  12a. [_] Bukumeka II A  12b. [_] Bukumeka II B  13. [_] Mahanga  14a. [_] Lwangoli  14b. [_] Buwangolo  15a. [_] Bumulahawasu  15b. [_] Bululsambu A  16a. [_] Musese Wapomokha  16b. [_] Musese Nakunuku  17a. [_] Wokukiri A  17a. [_] Wokukiri B  18a. [_] Bumahena  18b. [_] Nabikhoso  19. [_] Sisye cell B  20. [_] Doko cell C  21a. [_] Muti cell A  21b. [_] Muti cell B  21c. [_] Muti cell C  22a. [_] Mugisu cell A  22b. [_] Mugisu cell B  22c. [_] Kachumbala  23a. [_] Bujoroto cell B  23b. [_] Bujoroto cell D  24a.[_]Naksibisho cell A  24b.[_]Naksibisho cell B  24c. [_] Nagudi  25. [_] Other, specify | See recruitment interview |

## INTIAL SCREENING QUESTONS ABOUT THE MOTHER - INFANT PAIR

**To be confirmed, not to be asked literally**

EpiHandy p b (2)

| **1. QUESTION ENGLISH** | **2. ANSWER ENGLISH** | **3. SKIP/RULES** | **4. COLUMN FOR CODING** |
| --- | --- | --- | --- |

| 1. She is the mother of the baby | 1. [_] Yes  2. [_] No | **SKIP: If no,**  **Discontunue from SI** | EH: RB  VN: 1Yrb01 |
| --- | --- | --- | --- |
| 2. The baby is dead | 1. [_] Yes  2. [_] No | **SKIP: If yes,**  **Discontunue from SI**  **ADMINISTER INFANT VERBAL AUTOPSY FORM**  **(SEPARATE DOCUMENT)** | EH: RB  VN: 1Yrb02 |
| 3. The mother is dead | 1. [_] Yes  2. [_] No | **SKIP: If yes,**  **Discontunue from SI ADMINISTER MATERNAL VERBAL AUTOPSY FORM**  **(SEPARATE DOCUMENT)** | EH: RB  VN: 1Yrb03 |
| 4. The mother is away for other reasons | 1. [_] Yes  2. [_] No | **SKIP: If yes,**  **Discontunue from SI**  **ADMINISER MISSED VISIT/LOSS/TERMINATION FORM (SEPARATE DOCUMENT)** | EH: RB  VN: 1Yrb04 |
| 5. Planned revisit | **Date:** |  | EH: Date  VN: 1Yrb05 |
| 6.write down the name of the child | **-----------------------** |  | EH:RB  VN:name |

## SECTION I Infant feeding recalls

EpiHandy p. (e) 5

| **1. QUESTION ENGLISH** | **2. ANSWER ENGLISH** | **3.QUESTION**  **LUMASABA** | **4.ANSWER**  **LUMASABA** | **5. SKIP INSTRUCTION** | **6. COLUMN FOR CODING** |
| --- | --- | --- | --- | --- | --- |
| 1. Do you breastfeed ${name} $? | 1. [_] Yes ↓  2. [_] No | 1.Ununisakho${lisiina}$ | 1. [_] Ehh ↓  2. [_] Taawe | **SKIP: If yes, skip to q. 5** | EH: RB  VN: 1Yre01 |
| 2. Did you ever breastfed your child? | 1. [_] Yes *!5  2. [_] No ↓ | 2. Wanunitsitsakakho umwaana woowo? | 1. [_] Ehh *!5  2. [_] Taawe↓ | **SKIP: If no, skip to q. 4** | EH: RB  VN: 1Yre02 |
| 3. For how long did you breastfeed your child? | 1. Weeks: _______  2. [_] Do not know | 3**.** Imbuka shiina isi wayila nga ununisa umwaana woowo? | 1.Tsisabiti_______________  2. [_] Nakhumanya taawe | **< 1 week = 0**  **Report in full weeks** | EH: RB  a1: Num  a2: Nothing  VN: 1Yre03 |
| 4. What were your reasons for stopping to breastfeed/not breastfeed your child? | 1. [_] Work  2. [_] Education  3. [_] Illness, other than lactation problems  4. [_] Lactation problems  5. [_] Child not grow well  6. [_] Child crying a lot  7. [_] Not enough breastmilk  8. [_] No answer  9. [_] Advice/pressure from others  10. [_] Other, specify ________________ | 4**.** Shiina shinyene ishakyila wakamisa khununisa umwana woowo oba khuta khununisa taa? | 1. [_] Khuramba  2. [_] Khusoma  3. [_] Bulwale akhali biangafu bye khununisa  4.[_] Biangafu byekhununisa  5. [_] Umwaana atsowa bulayi taa  6. [_] Umwaana alila nabi  7. [_] Kamabele ikalimo kamala taa  8. [_] Mbawo  9. [_] Khubolela khwebabandu  10.[_] Ishindi,shiina……… |  | EH: CB  a9: Tx  VN: 1Yre04 |
| 5. Have you ever had any problem with your breast since your child was born? | 1. [_] Yes  2. [_] No ↓ | 5. Wabetsakakho ni bulwale bwosi khumabele khukhwama umwaana asalikha? | 1. [_] Ehh  2. [_] Taawe ↓ | **SKIP: If no, skip to the dietary 24-hour recall and q. 8** | EH: RB  VN: 1Yre05 |
| 6. What did you have? | 1. [_] Engorgement  2. [_] Cracked nipples  3. [_] Abcess  4. [_] Infection  5. [_] Operation  6. [_] Trauma  7. [_] Other, specify  _______________ | 6**.** Shaba shiina? | 1. [_] Khubimba  2. [_] Khukhwatikha busonga  3. [_] Libimba  4. [_] Bulwale  5. [_] Shisharo  6. [_] Khutsiniwa  7. [_] Ishindi shiina……… |  | EH: CB  VN: 1Yre06 |
| 7. How old was your baby when this occurred? | Weeks _______ | 7. Umwaana woowo aba nabukhulu shiina nga shakholekha? | Tsisabiti…………………. | **< 1 week = 0**  **Report in full weeks** | EH: Num (#)  VN: 1Yre07 |
| Since you stopped bleeding after the birth of your child:  8. Have you had menses after birth? | 1. [_] Yes  2. [_] No ↓ | Khukhwama wakama khutsya kamafukyi nga wasala:  8. Wafunatsyakakho kamafukyi nga wasala? | 1. [_] Ehh  2. [_]Taawe ↓ |  |  |
| 9. How old was your child when you had your first menses after birth? | Weeks _______ | 9. Umwana wowo aba alengana aryena isi watsila kamafukyi nga wamala khusala? | Tsisabiti----------------- |  |  |

**Dietary 24-hour recall:**

**English:**

I am now going to ask you questions about what you fed your baby from the time you woke up yesterday morning till you woke up this morning.

**Lumasaaba:**

Ari itsya khukhureba bireebo biambagana khubyeesi walisiile umwaana woowo khukwaama wenyukhile ingolobe kumutikhini khukhwoleesa shalee lo kumutikhini

| **1. QUESTION ENGLISH** | **2. ANSWER ENGLISH** | **3.QUESTION**  **LUMASABA** | **4.ANSWER**  **LUMASABA** | **5. SKIP INSTRUCTION** | **6. COLUMN FOR CODING** |
| --- | --- | --- | --- | --- | --- |
| 10. From the time you woke up yesterday morning till you woke up this morning did you breastfeed your baby? | 1. [_] Yes  2. [_] No ↓ | 10. Khukhwama nga wenyukhile ingolobe kumutikhini khukhwolesa shalelo kumutikhini wanunisilekho umwaana woowo? | 1. [_] Ehh  2. [_] Taawe ↓ | **SKIP: If no, skip to q. 11** | EH: Tx  VN: 1Yre08 |
| 11. From the time you woke up yesterday morning till you went to bed last night, how many times did you breastfeed? | ______(#(#)) | 11**.** Khukhwama nga wenyukhile ingolobe kumutikhini khukhwolesa isi watsile khukona, wanunisile kimilundu kyenga? | _____(#(#_) |  | EH: Num  VN: 1Yre09 |
| 12. From the time you went to bed last night till you woke up this morning, how many times did you breastfeed? | _______(#(#)) | 12. Khukhwama watsile khukona ingolobe khukhwolesa wenyukhile kumutikhini, wanunisile kimilundu kyenga? | _____(#(#_) |  | EH: Num  VN: 1Yre10 |

| 13. From the time you woke up yesterday morning till you woke up this morning:  Did you give any of the following items to the child? And if you did, will you please tell how many times you gave it? Did you give any:  1. Water Yes □ No □ Freq.[__]  2. Water with sugar or glucose Yes □ No □ Freq.[__]  3. Fruit juice Yes □ No □ Freq.[__]  4. Herbs Yes □ No □ Freq.[__]  5. Tea without milk Yes □ No □ Freq.[__]  6. Tea with milk Yes □ No □ Freq.[__]  7. Rice water Yes □ No □ Freq.[__]  8. Diluted cow’s milk Yes □ No □ Freq.[__]  9. Not diluted cow’s milk Yes □ No □ Freq.[__]  10. Infant formula Yes □ No □ Freq.[__]  11. Other powdered milk Yes □ No □ Freq.[__]  12. Dairy product like yoghurt, cream, sour milk  Yes □ No □ Freq.[__]  13. Goat’s milk Yes □ No □ Freq.[__]  14. Cereals, porridge, bread, fermented porridge  Yes □ No □ Freq.[__]  15. Fruits/vegetables Yes □ No □ Freq.[__]  16. Meat Yes □ No □ Freq.[__]  17. Fish Yes □ No □ Freq.[__]  18. Eggs Yes □ No □ Freq.[__]  19. Gripe water Yes □ No □ Specify  20. Non-prescribed medicine, specify  Yes □ No □ Freq.[__]  21. Prescribed medicine, specify Yes □ No □ Specify  22. Alcohol like beer or brew Yes □ No □ Freq.[__]  23. Other, Specify Yes □ No □ Freq.[__]  ____________________________________ | 13**.** Khukhwama nga niwenyukhile ingolobe khukhwolesa shalelo kumutikhini: Umwaana wamuwelekho byosi khubindu bino? Kale nga wamuwelekho, unyala wambolelakho kimilundu kyenga kyesi wamuwele? Wamuwakho shoosi khubino?  1. Kametsi Ehh □ Taawe □ Kimi.[__]  2. Kametsi ni sukali namwe kulukosi Ehh □ Taawe □ Kimi.[__]  3. Butunda Ehh □ Taawe □ Kimi.[__]  4. Kamalesi keshimali Ehh □ Taawe □ Kimi.[__]  5. Kyaayi umukhalu Ehh □ Taawe □ Kimi.[__]  6. Kyaayi uwe kamabele Ehh □ Taawe □ Kimi.[__]  7. Kamaetsi kemumukyele Ehh □ Taawe □ Kimi.[__]  8. Kamabele kengafu kalimo kametsi Ehh □ Taawe □ Kimi.[__]  9. Kamabele kengafu kakhalimo kametsi Ehh □ Taawe □ Kimi.[__]  10. Kamabele kebufu kebana Ehh □ Taawe □ Kimi.[__]  11. Kamabele kebufu Ehh □ Taawe □ Kimi.[__]  12. Kamabele kamaboyise, lubondo Ehh □ Taawe □ Kimi.[__]  13. Kamabele kembusi Ehh □ Taawe □ Kimi.[__]  14. Bilyo bye tsimunga,bubugyi namwe kumugati  Ehh □ Taawe □ Kimi.[__]  15. Kamatunda/tsinyanyi tsimali Ehh □ Taawe □ Kimi.[__]  16. Inyama Ehh □ Taawe □ Kimi.[__]  17. Iyeeni Ehh □ Taawe □ Kimi.[__]  18. Kamakyi Ehh □ Taawe □ Kimi.[__]  19. Kametsi kalimo kamalesi Ehh □ Taawe □ Kimi.[__]  20. Kamalesi kesi umusawu akhaweye, kabole  Ehh □ Taawe □ Kimi.[__]  21. Kamalesi kesi umusawu akhuweye, kabole Ehh □ Taawe □ Kimi.[__]  22. Shimesa shoosi nga bushela namwe inguli Ehh □ Taawe □ Kimi.[__]  23. Shiina ishindi, shibole Ehh □ Taawe □ Kimi.[__]  ____________________________________ | EH: List with Yes/No buttons  **NB: let all the specification options be Num! (99=DNK)**  VN: 1Yre11 |
| --- | --- | --- |

**Dietary** 1 Week recall

| **1. QUESTION ENGLISH** | **2. ANSWER ENGLISH** | **3.QUESTION**  **LUMASABA** | **4.ANSWER**  **LUMASABA** | **5. SKIP INSTRUCTION** | **6. COLUMN FOR CODING** |
| --- | --- | --- | --- | --- | --- |
| 14. Thinking one week back, have you breastfed your baby? | 1. [_] Yes  2. [_] No | 14.Nga wambasile musabiti indwela ibirile,wanunisakho umwaana woowo | 1. [_] Ehh  2. [_] Taawe |  | EH: RB  VN: 1Yre12 |

| 15. Now I am going to ask you if you gave the following items at all the last week ending yesterday morning.  Please answer yes if you gave it and no if you did not give it  1. Water Yes □ No □  2. Water with sugar or glucose Yes □ No □  3. Fruit juice Yes □ No □  4. Herbs Yes □ No □  5. Tea without milk Yes □ No □  6. Tea with milk Yes □ No □  7. Rice water Yes □ No □  8. Diluted cow’s milk Yes □ No □  9. Not diluted cow’s milk Yes □ No □  10. Infant formula Yes □ No □  11. Other powdered milk Yes □ No □  12. Dairy product like yoghurt, cream, sour milk  Yes □ No □  13. Goat’s milk Yes □ No □  14. Cereals, porridge, bread, fermented porridge  Yes □ No □  15. Fruits/vegetables Yes □ No □  16. Meat Yes □ No □  17. Fish Yes □ No □  18. Eggs Yes □ No □  19. Gripe water Yes □ No □  20. Non-prescribed medicine, specify  Yes □ No □  21. Prescribed medicine, specify Yes □ No □ Specify _______________  22. Alcohol like beer or brew Yes □ No □  23. Other, Specify Yes □ No □ Specify _______________  ____________________________________ | 15. Ari ndi khutsa khukhureba nga wawakho umwana khubindu bino musabiti indwela ibirile khukhwakama ingolobe, nga wamuwakho, ilamo uri ehh, nga wakhumuwakho, ilamo uri taawe.  1. Kametsi Ehh □ Taawe □  2. Kametsi ni sukali namwe kulukosi Ehh □ Taawe □  3. Butunda Ehh □ Taawe □  4. Kamalesi keshimali Ehh □ Taawe □  5. Kyaayi umukhalu Ehh □ Taawe □  6. Kyaayi uwe kamabele Ehh □ Taawe □  7. Kamaetsi kemumukyele Ehh □ Taawe □  8. Kamabele kengafu kalimo kametsi Ehh □ Taawe □  9. Kamabele kengafu kakhalimo kametsi Ehh □ Taawe □  10. Kamabele kebufu kebana Ehh □ Taawe □  11. Kamabele kebufu Ehh □ Taawe □  12. Kamabele kamaboyise, lubondo Ehh □ Taawe □  13. Kamabele kembusi Ehh □ Taawe □  14. Bilyo bye tsimunga,bubugyi namwe kumuga Ehh □ Taawe □  15. Kamatunda/tsinyanyi tsimali Ehh □ Taawe □  16. Inyama Ehh □ Taawe □  17. Iyeeni Ehh □ Taawe □  18. Kamakyi Ehh □ Taawe □  19. Kametsi kalimo kamalesi Ehh □ Taawe □  20. Kamalesi kesi umusawu akhaweye, kabole  Ehh □ Taawe □  21. Kamalesi kesi umusawu akhuweye, kabole Ehh □ Taawe □  22. Shimesa shoosi nga bushela namwe inguli Ehh □ Taawe □  23. Shiina ishindi,shibole Ehh □ Taawe □  _____________________ | EH: List with Yes/No buttons  **NB: let all the specification options be Num! (99=DNK)**  VN: 1Yre13 |
| --- | --- | --- |

**Dietary Recall since birth**

| 16. Now I am going to ask you if you ever have given the following to your baby and if you have done that, please tell us when you did that for the first time:  1 Water Yes □ No □ Wk __  2. Water with sugar or glucose Yes □ No □ Wk __  3. Fruit juice Yes □ No □ Wk __  4. Herbs Yes □ No □ Wk __  5. Tea without milk Yes □ No □ Wk __  6. Tea with milk Yes □ No □ Wk __  7. Rice water Yes □ No □ Wk __  8. Diluted cow’s milk Yes □ No □ Wk __  9. Not diluted cow’s milk Yes □ No □ Wk __  10. Infant formula Yes □ No □ Wk __  11. Other powdered milk Yes □ No □ Wk __  12. Dairy product like yoghurt, cream, sour milk  Yes □ No □ Wk __  13. Goat’s milk Yes □ No □ Wk __  14. Cereals, porridge, bread, fermented porridge  Yes □ No □ Wk __  15. Fruits/vegetables Yes □ No □ Wk __  16. Meat Yes □ No □ Wk __  17. Fish Yes □ No □ Wk __  18. Eggs Yes □ No □ Wk __  19. Gripe water Yes □ No □ Wk __  20. Non-prescribed medicine, specify Yes □ No □ Type: 1^st^ time:  21. Prescribed medicine, specify Yes □ No □ Type: 1^st^ time:  22. Alcohol like beer or brew Yes □ No □  23. Other, Specify Yes □ No □ Type: 1^st^ time: | 16. Ari indi khuutsya khureebakho nga wawetsakakho umwaaana woowo khubindu bino, atenga wamuwa, unyala wamboolelakho isi wamuwela kumulundi kunyoowa?:  1.Kameetsi Ehh □ Taawe □ Wk [__]  2.Kameetsi koosi ka sukaali namwe kulukosi Ehh □ Taawe □ Wk[__]  3. Butuunda Ehh □ Taawe □ Wk[__]  4. Kamalesi keshima Ehh □ Taawe □ Wk[__]  5. Kyaayi umukhalu Ehh □ Taawe □ Wk[__]  6. Kyaayi uwe kamabeele Ehh □ Taawe □ Wk[__]  7. Kameetsi kemukyele Ehh □ Taawe □ Wk[__]  8. Kamabeele kengafu kalimo kameetsi Ehh □ Taawe □ Wk[__]  9. Kamabeele kengafu kakhalimo kameetsi Ehh □ Taawe □ Wk[__]  10. Kamabeele kebuufu kebabaana Ehh □ Taawe □ Wk[__]  11. Kamabeele koosi kebuufu Ehh □ Taawe □ Wk[__]  12. Kamabeele kamaboyise,lubondo Ehh □ Taawe □ Wk[__]  13. Kamabeele kembusi Ehh □ Taawe □ Wk[__]  14. Bilyo bye tsimunga , bugyi , kumugati Ehh □ Taawe □ Wk[__]  15. Kamatuunda/tsinyinyi tsimali Ehh □ Taawe □ Wk[__]  16. Inyama Ehh □ Taawe □ Wk[__]  17. Inyeeni Ehh □ Taawe □ Wk[__]  18. Kamakyi Ehh □ Taawe □ Wk[__]  19. Kameetsi kalimo kamalesi Ehh □ Taawe □ Wk[__]  20. Kamalesi kesi umusawo akhawandikhile taa,kaboole Ehh □ Taawe □Kumulundu kwanyowa  21. Kamalesi kesi umasawo awandikhile,kaboole Ehh □ Taawe □ Kumulundu kwanyowa  22. Shimeesa shoosi nga busela, inguli Ehh □ Taawe □ Kumulundu kwanyowa  23. Nibayo Shishindi, shiboole Ehh □ Taawe □ Kumulundu kwanyowa  ______________________________________________ | EH: List with Yes/No buttons  **NB: let all the specification options be Num! (99=DNK)**  VN: 1Yre14 |
| --- | --- | --- |

## SECTION II Questions about leaving the child

**EpiHandy p. f** (6)

| **1. QUESTION ENGLISH** | **2. ANSWER ENGLISH** | **3.QUESTION LUMASAABA** | **4.ANSWER LUMASAABA** | **5. SKIP INSTRUCTION** | **6. COLUMN FOR CODING** |
| --- | --- | --- | --- | --- | --- |
| 1. Have you ever left your child since childbirth so that someone else has fed the child? | 1. [_] Yes  2. [_] No ↓ | 1. Walekhitsakakho umwaana woowo khukhwaama umusala numundi ukuundi wumuliisa*?* | 1. [_] Ehh  2. [_] Taawe ↓ | **SKIP: If no, skip to S III** | EH: RB  VN: 1Yrf01 |
| 2. What did the one taking care of your child feed last time? | 1. [_] Water based liquids  2. [_] Milk based liquids/semi-solid feeds  3. [_] Expressed breast milk from the mother  4. [_] Expressed breast milk from another woman, not the mother  5. [_] Do not know  6. [_] Other, specify ________ | 2. Uwalinda umwana woowo amuliisa shiina kumulundi ukwa sembayo? | 1.[_] Byekhunywa bye kamesi  2.[_] Byekhunywa bilimo kamabele/byekhulya bibyangu  3.[_] Libele likhamule  4.[_] Kamabele kama khamule uhkwama mu mukhasi ukundi  5.[_] Na khumanya  6.[_] Ibindi,biboole  **____________________** | **RULE: Tick off all that apply**  **Do not ask from the list, but probe from it.** | EH: CB  a6: Tx  VN: 1Yrf02 |
| 3. How often did it happen the last week that you had someone else to feed the child? | ____ Times/last week | 3. Isho shakholekha kimilundi kyenga musabiti iya sembayo isi walekhela umundu ukundi khulisa umwana? | ______Kimilundu/Isabiti iya weye |  | EH: Num  VN: 1Yrf03 |
| 4. How many times do you usually leave your baby per week? | ____ Times/week | 4. Umwana woowo umulekha kimilundi kyenga musabiti? | ______Kimilundu/Isabiti |  | EH: RB  VN: 21Yrf04 |

## SECTION III Bed Net, vaccination and micronutrients

**EpiHandy p. g (7)**

Now I am going to ask you questions which are related to your baby’s health:

| **1. QUESTION ENGLISH** | **2. ANSWER ENGLISH** | **3.QUESTION LUMASAABA** | **4.ANSWER LUMASAABA** | **5. SKIP INSTRUCTION** | **6. COLUMN FOR CODING** |
| --- | --- | --- | --- | --- | --- |
| 1. Does the baby sleep in your bed? | 1. [_] Yes  2. [_] No | 1. Umwaana ukona naye mu bulili bwo’wo? | 1. [_] Ehh  2. [_] Taawe |  | EH: RB  VN: 1Yrg01 |
| 2. Is the baby covered by a bednet at night? | 1. [_] Yes  2. [_] No | 2. Umwana arambisa khatimba khebulili mushiilo? | 1. [_] Ehh  2. [_] Taawe | **Both a separate net for the baby and a shared net with the mother qualifies for yes here** | EH: RB  VN: 1Yrg02 |
| 3. Has ${name} $ had any vaccinations? | 1. [_] Yes  2. [_] No ↓ | 3. ${lisiina} $ wafuna khugemebwa khwosi? | 1. [_] Ehh  2. [_] Taawe↓  3. [_] Nakhumanya taa. | **SKIP: If no, skip to q. 13** | EH: RB  VN: 1Yrg03 |
| 4. Has ${name} $ got the BCG vaccine? (mother’s answer)  (mother’s answer) | 1. [_] Yes  2. [_] No  3. [_] Do not know | 4. ${lisina} $ wafuna khugemebwa khwa BCG? (ukhwilamo khwa mayi) | 1. [_] Ehh  2. [_] Taawe  3. [_] Nakhumanya taa. | **Inform the mother that this was also asked last time**  **Given right upper arm(country specific)** | EH: List with Yes/No buttons  VN: 1Yrg04 |
| 5. Has ${name} $ got the first polio vaccine?(called polio o) (the first one) (mother’s answer) | 1. [_] Yes  2. [_] No  3. [_] Do not know | 5. ${lisina} $ wafuna khugemebwa khwa Polio khunyowa ¿(bulangibwa Polio 0) (khunyowa) (khukhwilamo khwamayi) | 1. [_] Ehh  2. [_] Taawe  3. [_] Nakhumanya taa. | **Inform the mother that this was also asked last time**  **Given as mouth drops** | EH: RB  VN: 1Yrg05 |
| 6. Has ${name} $ got the second polio vaccine (called polio 1)? | 1. [_] Yes  2. [_] No  3. [_] Do not know | 6. ${lisina} $ wafuna khugemebwa khwa Polio khwakhabili (bulangibwa polio 1)? | 1. [_] Ehh  2. [_] Taawe  3. [_] Nakhumanya taa. | **Inform the mother that this was also asked last time**  **Given as mouth drops** | EH: List with Yes/No buttons  VN: 1Yrg06 |
| 7. Has ${name} $ got the first triple vaccine(DPT)with the HepB and Hib 1?(Diphteria/Tetanus /Whooping Cough/Hepatitis B/Haemophius Influenza type B) | 1. [_] Yes  2. [_] No  3. [_] Do not know | 7. ${lisiina} $ wafuna khugemebwa khunyowa khulimo bikha bitaru (DPT) muli Hep B ni Hib 1? (Diptheria/Tetnus/Whooping  cough/Hepatitis B/Haemophius Influenzea type B) | 1. [_] Ehh  2. [_] Taawe  3. [_] Nakhumanya taa. | **Inform the mother that this was also asked last time**  **Usually given left upper thigh** | EH: RB  VN: 1Yrg07U |
| 8. Has ${name} $ got the third polio vaccine (called polio 2)? | 1. [_] Yes  2. [_] No  3. [_] Do not know | 8 ${lisina} $ wafuna khugemebwa khwa Polio khwekhataru (bulangibwa polio 2)? | 1. [_] Ehh  2. [_] Taawe  3. [_] Nakhumanya taa. | **Inform the mother that this was also asked last time**  **Given as mouth drops** | EH: RB  VN: 1Yrg08U |
| 9. Has ${name} $ got the second triple vaccine(DPT)with the HepB and Hib 1?(Diphteria/Tetanus /Whooping Cough/Hepatitis B/Haemophius Influenza type B) | 1. [_] Yes  2. [_] No  3. [_] Do not know | 9. ${lisiina} $ wafuna khugemebwa khwekhabiri khulimo bikha bitaru (DPT) muli Hep B ni Hib 1? (Diptheria/Tetnus/Whooping  cough/Hepatitis B/Haemophius Influenzea type B) | 1. [_] Ehh  2. [_] Taawe  3. [_] Nakhumanya taa. | **Inform the mother that this was also asked last time**  **Usually given left upper thigh** | EH: RB  VN: 1Yrg09U |
| 10. Has ${name} $ got the forth polio vaccine (called polio 3)? | 1. [_] Yes  2. [_] No  3. [_] Do not know | 10. ${lisina} $ wafuna khugemebwa khwa Polio khwekhane (bulangibwa polio 3)? | 1. [_] Ehh  2. [_] Taawe  3. [_] Nakhumanya taa. | **Given as mouth drops** | EH: RB  VN: 1Yrg10U |
| 11. Has ${name} $ got the third triple vaccine(DPT)with the HepB and Hib 1?(Diphteria/Tetanus /Whooping Cough/Hepatitis B/Haemophius Influenza type B) | 1. [_] Yes  2. [_] No  3. [_] Do not know | 11. ${lisiina} $ wafuna khugemebwa khwakhataru khulimo bikha bitaru (DPT) muli Hep B ni Hib 1? (Diptheria/Tetnus/Whooping  cough/Hepatitis B/Haemophius Influenzea type B) | 1. [_] Ehh  2. [_] Taawe  3. [_] Nakhumanya taa. | **Usually given left upper thigh** | EH: RB  VN: 1Yrg11U |
| 12. Has ${name} $ your baby got the measles vaccine? | 1. [_] Yes  2. [_] No  3. [_] Do not know | 12. Ne ${lisina} $ wafuna khugemebwa khwa mukusese? | 1. [_] Ehh  2. [_] Taawe  3. [_] Nakhumanya taa. |  | EH: RB  VN: 1Yrg12U |
| 13. Has ${name} $ ever got any Vitamin A capsule? | 1. [_] Yes  2. [_] No  3. [_] Do not know | 13. Ne ${lisiina}$ bamuwakho ikapiso iye khwongelakho bukali bwa Vitamin A? | 1. [_] Ehh  2. [_] Taawe  3. [_] Nakhumanya taa. | **Rule show the different types of Vit A capsules available in the area.** | EH: RB  VN: 1Yrg13U |

| Do not ask out loudly,but ask again to look at the child health card and:  14. Note down vaccinations given which is stated in the child health card.  Also note down if vitamin A is given: | 1. [_] BCG  2. [_] Polio O  3. [_] Polio 1  4. [_] DPT-HepB+Hib1  5. [_] Polio 2  6. [_] DPT- HepB+Hib2  7. [_] Polio 3  8. [_] DPT- HepB+Hib3  9. [_] Measles  10. [_] Vit A | Musabe mboola khulola khu lupapula lwe bye bulamu bwo mwaana:  14. Wandikha khugemebwa khwesi bawandikhile mu lupapula lwe bye bulamu | 1. [_] BCG *!6  2. [_] Polio O  3. [_] Polio 1  4. [_] DPT-HepB+Hib1  5. [_] Polio 2  6. [_] DPT- HepB+Hib2  7. [_] Polio 3  8. [_] DPT- HepB+Hib3  9. [_] Measles  10. [_] Vit A | **(P.S In Ugandan CHC it is written HebB and not HepB,tell this to the Ugandan DC)** | EH: List with yes/no buttons  VN: 1Yrg14U |
| --- | --- | --- | --- | --- | --- |
| 15. May I please see where they gave the BCG –vaccine at your baby’s right shoulder/upper arm? | Look for BCG-scar  1. [_] BCG-lession seen  2. [_] BCG-lession not seen | 15. Inyala nabonakho isi bakhupa khabiso kha BCG khwibeka lye kumukhono kumulayi kwo mwaana? | Look for BCG-lession  1. [_] BCG-lession seen  2. [_] BCG-lession not seen | **Halvor:SOP digital picture**  **Allow for BCG related wound or BCG scar** | EH: RB  VN: 1Yrg15U |
| 16. Now I would like to ask you about yourself.Since you gave birth: Have you taken any of these Vitamin A supplements? | 1. [_] Yes  2. [_] No  3. [_] Do not know | 16. Aari tsya khukhureba bikhuambakho: Khukhwama nga wasaala: Wamilakho khu kapisosi tsino tsye khwongelakho bukali bwe Vitamin A muma bele koowo? | 1. [_] Ehh  2. [_] Taawe  3. [_] Nakhumanya taa. | **Rule:DC show the different capsules** | EH: RB  VN: 1Yrg16U |
| 17. Since you gave birth to ${name} $ have you taken any of these iron tablets? | 1. [_] Yes,she identified that she had taken one or several of the iron tablets  2. [_] No,she confirmed that she had not taken any of the iron tablets  3. [_] She was not sure whether she had taken any of these iron tablets | 17. Khukhwama wasala ${lisina} $ wamilakho khamakalenda khe khwongela bukhali bwe kamafuki? | 1. [_] Eeh, wokesele ari wamila litwela oba makali busa khu makalenda khe kamafuki.  2. [_] Taawe, wokesele ari wakhumilatsakakho kamakelenda khe kamafuki ta.  3. [_] Bari amanya nga wamilakho kamakelenda khe kamafuki oba ta | **Show the different types of iron tablets available in the area**  **SKIP:If alternative 2 ticked off,skip question 10 and ask question 11** | EH: RB  VN: 1Yrg17U |
| 18. If yes, about how many iron tablets did you take during the whole pregnancy? | 1. [_] 1-10  2. [_] 11-30  3. [_] More than 30  4. [_] Don’t remember | 18. Nga ehh, wamilakho kamakalenda kongera bukali bwe kamafuki nga kenga mumbukha ye shisombo yosi? | 1. [_] 1-10  2. [_] 11-30  3. [_] Khufura mu 30  4. [_] Injebulila taa |  | EH: RB  VN: 1Yrg18 |
| 19. Did you take any other tablets containing iron during your pregnancy? If so can you please show me them? | 1. [_] No, did not take any other iron tablet  2. [_] Yes, and she showed tablets that contains iron  3. [_] said yes and showed tablets with unclear content or without iron  4. [_] said yes but did not have any tablets to show | 19. Wamilakho kamakelenda akandi khosi kalimo ibyongella bukali bwe kamafuki, nga shakholekha, unyala wakatsokesakho? | 1. [_] Taawe, nakhumilakho kamakalenda ka khwongella bukali bwe kamafuki ta  2. [_] Ehh, namilakho kamakalenda ka khwongella bukali bwe kamafuki  3. [_] Afukilile ne wokesa kamakelenda kakandi  4. [_] Afukilile ne sali ni kamakelenda ke khukhwokesa taa. | **Data collectors need to have a set of the most common iron tablets available so they can compare with those the woman show** | EH: RB  VN: 1Yrg19 |

## IV Morbidity, IV A Diarrhoea 24-h recall

**Epihandy p. h (8)**

**Diarrhoea 24-hour recall**

| **1. QUESTION ENGLISH** | **2. ANSWER ENGLISH** | **3.QUESTION LUMASAABA** | **4.ANSWER LUMASAABA** | **5. SKIP INSTRUCTION** | **6. COLUMN FOR CODING** |
| --- | --- | --- | --- | --- | --- |
| 1. From yesterday morning till this morning did ${name} $ have diarrhoea? | 1. [_] Yes  2. [_] No ↓ | 1. Ne ${lisiina} $ wabeleekho ni shiwalukho khukhwaama ingolobe kumutikhinyi?  Khukwolesa ari? | 1. [_] Ehh  2. [_] Taawe↓ | **SKIP: If no, skip to Diarrhoea 2 week recall**  **DC: Diarrhoe = loose or watery stools (1 to n times)** | EH: RB  VN: 1Yrh01 |
| 2. Did ${name} $ pass any watery stools? | 1. [_] Yes  2. [_] No | 2. Ne ${lisiina} $ awaalukhilekho biibi bilinga kameetsi? | 1. [_] Ehh  2. [_] Taawe | Watery stools= stools with no formed matter whatsoever | EH: RB  VN: 1Yrh02 |
| 3. How many loose or watery stools did ${name} $ pass? **R** | _______ (#(#)) | 3. Kimilundi kyenga kyeesi ${lisiina}$ a waalukhile biibi bilinga kameetsi? **R** | _______ (#(#)) |  | EH: Num  1Yrh03 |
| 4. Did any of the stools contain blood? | 1. [_] Yes  2. [_] No | 4. Manya khu biibi byosi byabelemo kamafukyi? | 1. [_] Ehh  2. [_] Taawe |  | EH: RB  VN: 1Yrh04 |
| 5. Were the stools of different consistency than before ${name} $ fell ill with diarrhoea? | 1. [_] Yes  2. [_] No | 5. Abe biibi ${lisiina}$ bishukhamo khufura nga ashiili khulwaala shiwalukho? | 1. [_] Ehh  2. [_] Taawe |  | EH: RB  VN: 1Yrh05 |
| 6. Did the illness interfere with ${name’s} $ ability to drink or eat? | 1. [_] Yes  2. [_] No | 6.Bulwale bwa karanganisa khunywa namwe khulya kho ${lisiina}$ | 1. [_] Ehh  2. [_] Taawe |  | EH: RB  VN: 1Yrh06 |
| 7. Did you seek treatment for ${name}$? | 1. [_] Yes  2. [_] No ↓ | 7. Ne ${lisiina} $ Wamuwetsalakho bukangi? | 1. [_] Ehh  2. [_] Taawe↓ | **SKIP: If no, skip to q. 9** | EH: RB  VN: 1Yrh07 |
| 8. Where did you go? | 1. [_] Relatives and friends  2. [_] Traditional healer  3. [_] Drugshop/ Pharmacy  4. [_] Government or private clinic/ surgery/community health centre including general practitioner  5. [_] The emergency/ outpatient department of a hospital  6. [_] Other, specify ________________ | 8.Watsya yena | 1[_] Balebe ni basaale  2.[_] Umusawo we shimali  3.[_] Khadukha khakulisa khamalesi  4.[_] Kikangilo lya gavumenti namwe lye mundu/isi babakila/likangilo lye shisitsa nga wakatilekho umusawo ukhola khubu balwale bwosi  5.[_] Likangilo linyala lya khuyeta nga wafunile buwangafu bwosi  6.[_] Akhundi waye,boola  __________________ |  | EH: CB  a6: Tx  VN:  1Yrh08 |
| 9. Was the child admitted to a hospital? | 1. [_] Yes  2. [_] No ↓ | 9. Ne umwana bamuwa shitanda mwikangilo? | 1. [_] Ehh  2. [_] Taawe↓ | **SKIP: If no, skip to Diarrhoea 2 week recall** | EH: RB  VN: 1Yrh09 |
| 10. Please give name of hospital? | 1. [_] Mbale Main hospital  2. [_] Bududa Hospital  3. [_] Busiu  4. [_] Bushacori  5. [_] SIRA  6. [_] Bufumbo  7. [_] Mission  8. [_] JOY  9. [_] Cure Hospital  10. [_] Ahamedia  11. [_] St. Martin  12. [_] Other, specify ________________ | 10. Kale khubolelekho lisiina lye likangilo? | 1. [_] Mbale Main hospital  2. [_] Bududa Hospital  3. [_] Busiu  4. [_] Bushicori  5. [_] SIRA  6. [_] Bufumbo  7. [_] Mission  8. [_] JOY  9. [_] Cure Hospital  10. [_] Ahamediya  11. [_] St. Martins  12. [_] Lindi shiina, liboole |  | EH: RB  **VN :**  **1Yrh10U** |
| 11. Was this the nearest health unit? | 1. [_] Yes  2. [_] No ↓ | 11. Liino likangilo lyaba lyaambi khuminya? | 1. [_] Ehh  2. [_] Taawe↓ | **SKIP: If no, skip to Diarrhoea 2 week recall** | EH: RB  VN: 1Yrh11 |
| 12. Why did you go there? | 1. [_] Health cervices better than at the nearest health unit  2. [_] Transport was available  3. [_] The nearest health unit is more expensive than the one I went to  4. [_] I wanted to go to the biggest hospital I can afford  5. [_] I do not trust the people at the nearest health unit  6. [_] Other, specify | 12. Lwashi watsya mwi kangilo lyo? | 1.[_] Bakhola bulayi khuminya likangilo lye aambi  2.[_] Ingenda yabawo  3.[_] Likangilo lye ambi basaba tsinusu ngali khuminya isi natsya  4.[_] Nakanah khutsya mwikangilo liminya bukali mwesi inyala khusasula tsinusu  5.[_] Sinikakasa abandu bali mwikangilo lye ambi  6.[_] Shindi shiina,shiboole_____ |  | EH: CB  a6: Tx  VN:  1Yrh12 |

## Diarrhoea 2 week recall

**E**pihandy p. i (9)

| **1. QUESTION ENGLISH** | **2. ANSWER ENGLISH** | **3.QUESTION LUMASAABA** | **4.ANSWER LUMASAABA** | **5. SKIP INSTRUCTION** | **6. COLUMN FOR CODING** |
| --- | --- | --- | --- | --- | --- |
| 1. During the last two weeks that ended yesterday morning, did ${name}$ have diarrhoea? | 1. [_] Yes  2. [_] No ↓ | 1. Ne ${lisiina}$ wabeleekho ni shiwalukho musabiti tsibili tsibirire khukhwakama ingolobe? | 1. [_] Ehh  2. [_] Taawe↓ | **SKIP: If no, skip to 24-hour recall for pneumonia/ALRI questions**  **DC: Diarrhoe = loose or watery stools (1 to n times)** | EH: RB  VN:1Yri01 |
| 2. Did ${name} $ pass any watery stools? | 1. [_] Yes  2. [_] No | 2. Ne ${lisiina} $ awaalukhilekho biibi bilinga kameetsi? | 1. [_] Ehh  2. [_] Taawe | Watery stools= stools with no formed matter whatsoever | EH: RB  VN: 1Yri02 |
| 3. The day ${name}$ had most loose or watery stools, how many loose or watery stools did ${name}$ pass? **R** | _______ (#(#)) | 3. Kimilundi kyenga kyeesi ${lisiina}$ a waalukhile biibi bilinga kameetsi? **R** | _______ (#(#)) | **RULE: Write 99 for number of stools if informant does not remember** | EH: Num  VN: 1Yri03 |
| 4. Did any of the stools contain blood? | 1. [_] Yes  2. [_] No | 4. Manya khu biibi byosi byabelemo kamafukyi? | 1. [_] Ehh  2. [_] Taawe |  | EH: RB  VN: 1Yri04 |
| 5. Were the stools of different consistency than before ${name}$ fell ill with diarrhoea? | 1. [_] Yes  2. [_] No | 5. Abe biibi ${lisiina}$ bishukhamo khufura nga ashiili khulwaala shiwalukho? | 1. [_] Ehh  2. [_] Taawe |  | EH: RB  VN: 1Yri05 |
| 6. Did the illness interfere with ${name’s}$ ability to drink or eat? | 1. [_] Yes  2. [_] No | 6.Bulwale bwa karanganisa khunywa namwe khulya khwa ${lisiina}$ | 1. [_] Ehh  2. [_] Taawe |  | EH: RB  VN: 1Yri06 |
| 7. Did you seek treatment for ${name}$? | 1. [_] Yes  2. [_] No ↓ | **7**. Ne ${lisiina}$ wamuwetsalakho bukangi? | 1. [_] Ehh  2. [_] Taawe↓ |  | EH: RB  VN: 1Yri07 |
| 8. Where did you go? | 1. [_] Relatives and friends  2. [_] Traditional healer  3. [_] Drugshop/ Pharmacy  4. [_] Government or private clinic/ surgery/community health centre including general practitioner  5. [_] The emergency/ outpatient department of a hospital  6. [_] Other, specify ________________ | 8.Watsya yena | 1[_] Balebe ni basaale  2.[_] Umusawo we shimali  3.[_] Khadukha khakulisa khamalesi  4.[_] Kikangilo lya gavumenti namwe lye mundu/isi babakila/likangilo lye shisitsa nga wakatilekho umusawo ukhola khubu balwale bwosi  5.[_] Likangilo linyala lya khuyeta nga wafunile buwangafu bwosi  6.[_] Akhundi waye,boola  **__________________** |  | EH: CB  a6: Tx  VN:  1Yri08 |
| 9. Was the child admitted to a hospital? | 1. [_] Yes  2. [_] No ↓ | 9. Ne umwana bamuwa shitanda mwikangilo? | 1. [_] Ehh  2. [_] Taawe↓ | **SKIP: If no, skip to q.13** | EH: RB  VN: 1Yri09 |
| 10. Please give name of hospital? | 1. [_] Mbale Main hospital  2. [_] Bududa Hospital  3. [_] Busiu  4. [_] Bushacori  5. [_] SIRA  6. [_] Bufumbo  7. [_] Mission  8. [_] JOY  9. [_] Cure Hospital  10. [_] Ahamadiya  11. [_] St. Martin  12. [_] Other, specify ________________ | 5. Kale khubolelekho lisiina lye likangilo? | 1. [_] Mbale Main hospital  2. [_] Bududa Hospital  3. [_] Busiu  4. [_] Bushacori  5. [_] SIRA  6. [_] Bufumbo  7. [_] Mission  8. [_] JOY  9. [_] Cure Hospital  10. [_] Ahamadiya  11. [_] St. Martin  12. [_] Lindi shiina, liboole ________________ |  | EH: RB  VN: 24i10  **VN:1Yri10U** |
| 11. Was this the nearest health unit? | 1. [_] Yes  2. [_] No ↓ | 11. Liino likangilo lyaba khambi khuminya? | 1. [_] Ehh  2. [_] Taawe↓ | **SKIP: If no, skip to Pneumonia 24-h recall** | EH: RB  VN: 1Yri11 |
| 12. Why did you go there? | 1. [_] Health cervices better than at the nearest health unit  2. [_] Transport was available  3. [_] The nearest health unit is more expensive than the one I went to  4. [_] I wanted to go to the biggest hospital I can afford  5. [_] I do not trust the people at the nearest health unit  6. [_] Other, specify | 12. Lwashi watsya mwi kangilo lyo? | 1.[_] Bakhola bulayi khuminya likangilo lye aambi  2.[_] Ingenda siyabawo  3.[_] Likangilo lye ambi basaba tsinusu tsingali khuminya isi natsya  4.[_] Nakanah khutsya mwikangilo liminya bukali mwesi inyala khusasula tsinusu  5.[_] Sinikakasa abandu bali mwikangilo lye ambi  6.[_] Shindi shiina,shiboole_____ |  | EH: CB  a6: Tx  VN:1Yri12 |
| 13. ALT 1: How many days did the diarrhoea last/  ALT 2: How many days has the diarrhoea lasted? | 1. [_] Finished: Duration in days __________  2. [_] Not finished: Duration in days: ____________ | 13. ALT 1: Awalukhila shiwalukho tsinakhu tsenga?  ALT2: Shiwalukho shamalile tsinakhu tsenga? | 1. [_] Shakama: Munakkhu tsenga __________  2. [_] Shakhwakama taa: Tsinakhu tsenga  ……………………. | **Make skip instruction so that you ask alt 1 for a neg. 24 h recall and alt 2 for a post 24 h recall** | EH: RB  a1: Num  a2: Num  VN: 1Yri13 |
| 14. During this period of illness you have described, did you change the way you were feeding your child in any way? | 1. [_] Yes  2. [_] No ↓ | 14. Mumbuka iye bulwaale esi ukanikhilekho, washusakho khuliiisa kho umwana woowo musaambo yooosi? | 1. [_] Ehh  2. [_] Taawe↓ | **SKIP: If no, skip to q. 16** | EH: RB  VN: 1Yri14 |
| 15. In which way? | 1. [_] Stopped breast feeding  2. [_] Stopped non-human milk  3. [_] Stopped other liquids  4. [_] Stopped solid foods  5. [_] Only breast fed at night  6. [_] Began giving other liquids  7. [_] Began giving solid foods  8. [_] Other, specify | 15. Munjeli shi? | 1.[_] Nakamisa khununisa  2.[_] Nakamisa kamabele kakhali kebabandu  3.[_] Nakamisa bye khunywa ibindi  4.[_] Nakamisa bye khulya  5.[_] Nanunisa shilo shonyene  6.[_] Na ntandikha khuwa bye khulya ibindi  7.[_] Na ntandikha khuwa bye khulya  8.[_] Ishindi shiina,shiboole |  | EH: CB  a8: Tx  VN: 1yri15 |
| 16. During the period of illness did you feed your baby more often, more seldom than or just as often as before the illeness started? | 1. [_] More often  2. [_] More seldom than before the illness started  3. [_] Did not change feeding frequency. | 16. Mumbuka nga mwana woowo alwala, wamununisa bulikhasela namwe wiyongera bussa nga inyuma nga ashili khulwala? | 1.[_] Bulikhasela  2.[_] Bulikhasela namwe khufurisakho nga umwana ashili khulwala  3.[_] Sinashusa kho kimilundi kyekhulisa |  | EH: RB  VN: 1Yri17 |
| **Recall on persistent diarrhoea** |  |  |  |  |  |
| 1. Since last visit, (which was when the baby was 12 weeks (3 months old): Has your child had diarrhoea which lasted 2 weeks or longer? | 1. [_] Yes, specify  2. [_] No | 17. Khukhwama nakhukyeniyi nga umwaana ali kyimwesi kyitaru, umwaana walwala shiwalukho she tsisabiti tsibili oba khufura? | 1. [_] Ehh  2. [_] Taawe | **Rule: The DC specifies when the last visit was, if 12 weeks was lost, and then ask since 6 weeks and so forth.**  **If no,skip to next section** | EH: RB  VN: 1yrn01 |
| 2.Would you please tell me how old ${name}$ was when the last such episode started/ | ANSWER GIVEN IN:  1……..(#(#)days  or  2…….(#(#)wks  or  3……..(#(#)mo | 2. Unyala wambolela kho bukhulu bwa ${lisiina}$ isi alwalila shiwalukho kumulundi kukwasembayo. | SHESI ILILEMO:  1……(#(#)tsinakhu  oba  2…….(#(#)tsisabiti  oba  3……..(#(#)kyimyesi |  | EH:RB  VN:1Yrn02 |
| 3.Did you seek treatment for ${name}$ | 1. [_] Yes  2. [_] No | 3. Wayetselakho ${lisiina}$ bukangi? | 1. [_] Ehh  2. [_] Taawe | **If no skip to next section** | EH:RB  VN:1Yrn03 |
| 4. Where did you go? | 1. [_] Relatives and friends  2. [_] Traditional healer  3. [_] Drugshop/ Pharmacy  4. [_] Government or private clinic/ surgery/community health centre including general practitioner  5. [_] The emergency/ outpatient department of a hospital  6. [_] Other, specify ________________ | 8.Watsya yena | 1[_] Balebe ni basaale  2.[_] Umusawo we shimali  3.[_] Khadukha khakulisa khamalesi  4.[_] Kikangilo lya gavumenti namwe lye mundu/isi babakila/likangilo lye shisitsa nga wakatilekho umusawo ukhola khubu balwale bwosi  5.[_] Likangilo linyala lya khuyeta nga wafunile buwangafu bwosi  6.[_] Akhundi waye,boola  **__________________** |  | EH:CB  VN:1Yrn04 |
| 5. Was ${name}$ admitted to hospital? | 1. [_] Yes  2. [_] No | 5. Ne ${lisiina}$ bamuwa shitanda mwikangilo? | 1. [_] Ehh  2. [_] Taawe | **If no skip to next section** | EH:RB  VN:1Yrn05 |
| 6. Please give name of hospital? | 1. [_] Mbale Main hospital  2. [_] Bududa Hospital  3. [_] Busiu  4. [_] Bushacori  5. [_] SIRA  6. [_] Bufumbo  7. [_] Mission  8. [_] JOY  9. [_] Cure Hospital  10. [_] Ahamadiya  11. [_] St. Martin  12. [_] Other, specify ________________ |  | 1. [_] Mbale Main hospital  2. [_] Bududa Hospital  3. [_] Busiu  4. [_] Bushacori  5. [_] SIRA  6. [_] Bufumbo  7. [_] Mission  8. [_] JOY  9. [_] Cure Hospital  10. [_] Ahamadiya  11. [_] St. Martin  12. [_] Other, specify ________________ |  |  |

## IV B ALRI/pneumonia

## Pneumonia 24-hour recall

**EpiHandy p. j (10)**

| **1. QUESTION ENGLISH** | **2. ANSWER ENGLISH** | **3.QUESTION LUMASAABA** | **4.ANSWER LUMASAABA** | **5. SKIP INSTRUCTION** | **6. COLUMN FOR CODING** |
| --- | --- | --- | --- | --- | --- |
| 1. From yesterday morning till this morning, did ${name}$ have cough? | 1. [_] Yes  2. [_] No | 1. Khukhwaama kumutikhini kwe ngolobe khukholesa ari ${lisiina}$ wabelekho ni shikhololo? | 1. [_] Ehh  2. [_] Taawe |  | EH: RB  VN: 1Yrj01 |
| 2. From yesterday morning till this morning, did ${name}$ have fast or difficult breathing? | 1. [_] Yes  2. [_] No ↓ | 2. Khukhwaama kumutikhini ingolobe khukholesa ari ${lisiina}$ wabelekho nikhuwela khwaamangu namwe buwangafu mu khuwela? | 1. [_] Ehh  2. [_] Taawe ↓ | **SKIP: If no q. 1 and 2 skip to Pneumonia 2 week recall** | EH: RB  VN: 1Yrj02 |
| 3. Did the illness interfere with ${name}$ ability to drink or eat? | 1. [_] Yes  2. [_] No | 3.Ne bulwale bwa karanganisa khunywa namwe khulya khwa ${lisiina}$ | 1. [_] Ehh  2. [_] Taawe |  | EH: RB  VN: 1Yrj03 |
| 4. Was ${name}$ admitted to a hospital for the illness? | 1. [_] Yes  2. [_] No ↓ | 4. Ne ${lisiina}$ bamuwelekho shitanda mudwaliro? | 1. [_] Ehh  2. [_] Taawe↓ | **SKIP: If no, skip to Pneumonia 2 week recall** | EH: RB  VN: 1Yrj04 |
| 5. Please give name of hospital? | 1. [_] Mbale Main hospital  2. [_] Bududa Hospital  3. [_] Busiu  4. [_] Bushacori  5. [_] SIRA  6. [_] Bufumbo  7. [_] Mission  8. [_] JOY  9. [_] Cure Hospital  10. [_] Ahamadiya  11. [_] St. Martin  12. [_] Other, specify ________________ | 5. Kale khubolelekho lisiina lye likangilo? | 1. [_] Mbale Main hospital  2. [_] Bududa Hospital  3. [_] Busiu  4. [_] Bushicori  5. [_] SIRA  6. [_] Bufumbo  7. [_] Mission  8. [_] JOY  9. [_] Cure Hospital  10. [_] Ahamadiya  11. [_] St. Martins  12. [_] Lindi shiina, liboole ________________ |  | EH: RB  VM: 24j05  **VN :1Yrj05U** |
| 6. Was this the nearest health unit? | 1. [_] Yes  2. [_] No ↓ | 6. Liino likangilo lyaba khambi khuminya? | 1. [_] Ehh  2. [_] Taawe ↓ | **SKIP: If yes, skip to Pneumonia 2 week recall** | EH: RB  VN: 24j06 |
| 7. Why did you go there? | 1. [_] Health cervices better than at the nearest health unit  2. [_] Transport was available  3. [_] The nearest health unit is more expensive than the one I went to  4. [_] I wanted to go to the biggest hospital I can afford  5. [_] I do not trust the people at the nearest health unit  6. [_] Other, specify | 7. Lwashi watsya mwi kangilo lyo? | 1.[_] Bakhola bulayi khuminya likangilo lye aambi  2.[_] Ingenda yabawo  3.[_] Likangilo lye ambi basaba tsinusu tsingali khuminya isi natsya  4.[_] Nakanah khutsya mwikangilo liminya bukali mwesi nyala khusasula tsinusu  5.[_] Sinikakasa abandu bali mwikangilo lye ambi  6.[_] Shindi shiina,shiboole_____ |  | EH: CB  a6: Tx  VN:1Yrj07 |

## Pneumonia 2 Week recall

EpiHandy p. k (11)

| **1. QUESTION ENGLISH** | **2. ANSWER ENGLISH** | **3.QUESTION LUMASAABA** | **4.ANSWER LUMASAABA** | **5. SKIP INSTRUCTION** | **6. COLUMN FOR CODING** |
| --- | --- | --- | --- | --- | --- |
| 1. During the last two weeks that ended yesterday morning, did ${name}$ have cough? | 1. [_] Yes  2. [_] No | 1. Ne ${lisiina}$ wabeleekho ni shikhololo musabiti tsibili tsibirire khukhwakama ingolobe? | 1. [_] Ehh  2. [_] Taawe |  | EH: RB  VN: 1Yrk01 |
| 2. During the last two weeks that ended yesterday morning, did ${name}$ have fast or difficult breathing? | 1. [_] Yes  2. [_] No ↓ | 2. Musabiti tsibili tsitsa kamile ingolobe ${lisiina}$ wabelekho nikhuwela khwaamangu namwe buwangafu mu khuwela? | 1. [_] Ehh  2. [_] Taawe ↓ | **SKIP: If no q. 1 and 2 skip to hospitalization section** | EH: RB  VN: 1Yrk02 |
| 3. Did the illness interfere with ${name’s}$ ability to drink or eat? | 1. [_] Yes  2. [_] No | 3.Ne bulwale bwa karanganisa khunywa namwe khulya khwa ${lisiina}$ | 1. [_] Ehh  2. [_] Taawe |  | EH: RB  VN: 1Yrk03 |
| 4. Was ${name}$ admitted to a hospital for the illness? | 1. [_] Yes  2. [_] No ↓ | 4. Ne ${lisiina}$ bamuwelekho shitanda mudwaliro? | 1. [_] Ehh  2. [_] Taawe↓ | **SKIP: If no, skip to q. 8** | EH: RB  VN: 1Yrk04 |
| 5. Please give name of hospital? | 1. [_] Mbale Main hospital  2. [_] Bududa Hospital  3. [_] Busiu  4. [_] Bushacori  5. [_] SIRA  6. [_] Bufumbo  7. [_] Mission  8. [_] JOY  9. [_] Cure Hospital  10. [_] Ahamadiya  11. [_] St. Martin  12. [_] Other, specify ________________ | 5. Kale khubolelekho lisiina lye likangilo? | 1. [_] Mbale Main hospital  2. [_] Bududa Hospital  3. [_] Busiu  4. [_] Bushicori  5. [_] SIRA  6. [_] Bufumbo  7. [_] Mission  8. [_] JOY  9. [_] Cure Hospital  10. [_] Ahamadiya  11. [_] St. Martins  12. [_] Lindi shiina, liboole ________________ |  | EH: RB  VN: 24k05  **VN:1Yrk05U** |
| 6. Was this the nearest health unit? | 1. [_] Yes  2. [_] No ↓ | 6. Liino likangilo lyaba khambi khuminya? | 1. [_] Ehh  2. [_] Taawe ↓ | **SKIP: If no, skip to q. 8** | EH: RB  VN: 1Yrk06 |
| 7. Why did you go there? | 1. [_] Health cervices better than at the nearest health unit  2. [_] Transport was available  3. [_] The nearest health unit is more expensive than the one I went to  4. [_] I wanted to go to the biggest hospital I can afford  5. [_] I do not trust the people at the nearest health unit  6. [_] Other, specify | 7. Lwashi watsya mwi kangilo lyo? | 1.[_] Bakhola bulayi khuminya likangilo lye aambi  2.[_] Ingenda yabawo  3.[_] Likangilo lye ambi basaba tsinusu tsingali khuminya isi natsya  4.[_] Nakanah khutsya mwikangilo liminya bukali mwesi inyala khusasula tsinusu  5.[_] Sinkakasa abandu bali mwikangilo lye ambi  6.[_] Shindi shiina,shiboole_____ |  | EH: CB  a6: Tx  VN: 1Yrk07 |
| 8. During this period of illness you have described, did you change the way you were feeding your child in any way? | 1. [_] Yes  2. [_] No ↓ | 8. Mumbuka iye bulwaale esi ukanikhilekho, washusakho khuliiisa khwo umwana woowo musaambo yooosi? | 1. [_] Ehh  2. [_] Taawe↓ | **SKIP: If yes, skip to q.10** | EH: RB  VN: 1Yrk8 |
| 9. In which way? | 1. [_] Stopped breast feeding  2. [_] Stopped non-human milk  3. [_] Stopped other liquids  4. [_] Stopped solid foods  5. [_] Only breast fed at night  6. [_] Began giving other liquids  7. [_] Began giving solid foods  8. [_] Other, specify | 9. Munjeli shi? | 1.[_] Nakamisa khununisa  2.[_] Nakamisa kamabele kakhali kebabandu  3.[_] Nakamisa bye khunywa ibindi  4.[_] Nakamisa bye khulya  5.[_] Nanunisa shilo shonyene  6.[_] Na ntandikha khumuwa bye khunywa ibindi  7.[_] Na ntandikha khumuwa bye khulya  8.[_] Ishindi shiina,shiboole |  | EH: CB  a8: Tx  VN: 1Yrk9 |
| 10. During the period of illness did you feed your baby more often, more seldom than or just as often as before the illeness started? | 1. [_] More often  2. [_] More seldom than before the illness started  3. [_] Did not change feeding frequency. | 10. Mumbuka nga mwana woowo alwala, wamununisa bulikhasela namwe wiyongera bussa nga inyuma nga ashili khulwala? | 1.[_] Bulikhasela  2.[_] Bulikhasela namwe khufurisakho nga umwana ashili khulwala  3.[_] Sinashusa kho kimilundi kyekhulisa |  | EH: RB  VN: 1Yrk10 |

## IV C Hospitalizations

**EpiHandy p. l (12)**

| **1. QUESTION ENGLISH** | **2. ANSWER ENGLISH** | **3.QUESTION LUMASAABA** | **4.ANSWER LUMASAABA** | **5. SKIP INSTRUCTION** | **6. COLUMN FOR CODING** |
| --- | --- | --- | --- | --- | --- |
| 1. Since birth has ${name}$ ever been admitted to hospital? | 1. [_] Yes  2. [_] No ↓ | 1. Ne ${lisiina}$ bamuwakho shitaanda mwikangilo khukhwama umusala? | 1. [_] Ehh  2. [_] Taawe ↓ | **SKIP: If no, skip to S V** | EH: RB  VN: 1Yrl01 |
| 2. How many times has ${name}$ been admitted to hospital? | _____ (#(#)) | 2. Kimilundi kyenga kyeesi ${lisiina}$ abeela khu shintaanda mwikangilo? | _____ (#(#)) |  | EH: Num  VN: 1Yrl02 |
| 3. How old in weeks was your baby (each time) when he/she was in hospital? | 1. [_] 1^st^ time ______  2. [_] 2^nd^ time ______  3. [_] 3rd time _____  4. [_] 4th time ______  5. [_] 5^th^ time ______  6. [_] 6^th^ time ______  7. [_] 7^th^ time ______  8. [_] 8^th^ time ______ | 4. Yaba umwaana ali ni tsisabiti tsenga uli kumulundu kwesi bamuwela shitanda? | 1. [_] Kumulundu kunyowa ______  2. [_] Kumulundu kwakhabili______  3. [_] Kumulundu kwakhataru _____  4. [_] Kumulundu kwakhane ______  5. [_] Kumulundu kwakharano ______  6. [_] Kumulundu kwakhasesaba ______  7. [_] Kumulundu kwekumusavu ______  8. [_] Kumulundu kweshinane………….. | **RULE: Tick off all that apply** | EH: CB  a1: Num  a2: Num  a3: Num etc.  Allows for DNK for alternatives  VN: 1Yrl03 |
| 4. For how many days was ${name}$ (each time) in hospital? | 1. [_] 1^st^ time ______  2. [_] 2^nd^ time ______  3. [_] 3rd time _____  4. [_] 4th time ______  5. [_] 5^th^ time ______  6. [_] 6^th^ time ______  7. [_] 7^th^ time ______  8. [_] 8^th^ time ______ | 4. Tsinakhu tsenga tsesi ${lisiina}$ amala mudwali uli kumulundi? | 1. [_] Kumulundu kunyowa ______  2. [_] Kumulundu kwakhabili______  3. [_] Kumulundu kwakhataru _____  4. [_] Kumulundu kwakhane ______  5. [_] Kumulundu kwakharano ______  6. [_] Kumulundu kwakhasesaba ______  7. [_] Kumulundu kwekumusavu ______  8. [_] Kumulundu kweshinane………….. |  | EH: CB  a1: Num  a2: Num  a3: Num etc.  VN: 1Yrl04 |
| 5. What was the reason ${name}$ was in the hospital each time: | 1. [_] 1^st^ time ______  2. [_] 2^nd^ time ______  3. [_] 3rd time _____  4. [_] 4th time ______  5. [_] 5^th^ time ______  6. [_] 6^th^ time ______  7. [_] 7^th^ time ______  8. [_] 8^th^ time ______ | 5. Yaba bulwale shiina bwesi ${lisiina}$ afunilakho shitanda mudwaliro uli kumulundu: | 1. [_] Kumulundu kunyowa ______  2. [_] Kumulundu kwakhabili______  3. [_] Kumulundu kwakhataru _____  4. [_] Kumulundu kwakhane ______  5. [_] Kumulundu kwakharano ______  6. [_] Kumulundu kwakhasesaba ______  7. [_] Kumulundu kwekumusavu ______  8. [_] Kumulundu kweshinane………….. | **RULE: ENTER THE CORRECT NUMBER FROM THE LIST BELOW**  1 = Diarrhoea  2 = Pneumonia/ “Cought and difficult breathing”  3 = Malaria  4 = Accident  5 = Specify, what ___________ | EH: CB  a1: Tx  a2: Tx  a3: Tx etc.  Allows for DNK for alternatives  VN: 1Yrl05 |

**V Complimentary feeding**

| **1. QUESTION ENGLISH** | **2. ANSWER ENGLISH** | **3.QUESTION LUMASAABA** | **4.ANSWER LUMASAABA** | **5. SKIP INSTRUCTION** | **6. COLUMN FOR CODING** |
| --- | --- | --- | --- | --- | --- |
| 1. Did you give *<NAME>*anything other than breast milk from this time yesterday until now? | 1. [_] Yes  2. [_] No ↓ | 1. Waweyekho <LIISINA> ishindu ishindi shosi akhali libele ukwama isawa nga eyi ingolobe ukholesa ari? | 1.[_]Eeh  2.[_]Taawe ↓ | **SKIP: If no, skip to S VI** | EH: RB  VN: 1Yrn01 |
| 2. What kind of food did you give? | 1. [_] Specially prepared for the baby  2. [_] Adult/family food | 2. Wawa bilyo shiina? | 1.[_] Byesi barengehele burawa umwana  2. [_] Bakhulu bemungo byesi balyakho |  | EH: RB  VN: 1Yrn02 |
| 3. What did you use to give *<NAME>* something to eat yesterday or last night? | 1. [_]Cup with spout  2. [_]Bottle with nipple  3. [_]Spoon  4. [_]Hand  5. [_]Don’t Know  6. [_] Other specify________ | 3. Warambisa shiina khuwa <LIISINA> bye khulya angolobe oba mushilo? | 1. [_] Shikombe shilikho shifunikho?  2. [_] Ikhupa ye khununa  3. [_] Shijikho  4. [_] Kumukhono  5. [_] Na khumanya taawe  6.[_] Bindi shiina, biboole__________ | **RULE: Tick off all that apply** | EH: CB  Allows for DNK for alternatives  VN: 1Yrn03 |
| 4. Was any of the following added to the foods or liquids *<NAME>* ate yesterday or last night? | 1. Sugar  [_] Yes [_] No  2.Cooking Oil/butter  [_] Yes  [_] No  3.Milk  [_] Yes  [_] No | 4. Iliwo khubino byesi wayongelekho mu bilyo oba byekhunya byesi <LIISINA> alile ingolobe oba mushiilo? | 1. Sukhali  [_] Eeh  [_] Taawe  2. Buuto/Kumusigo  [_] Eeh  [_] Taawe  3. Kamabelle  [_] Eeh  [_] Taawe | **RULE: Tick off all that apply** | EH: CB  a1: RB  a2: RB  a3: RB  VN: 1Yrn04 |
| 5. Do you give any food items to *<NAME>* that you have specifically bought from the shop or market for her/him? | 1. [_] Yes  2. [_] No | 5. Uwakho <LIISINA> byekhulya byosi bya burawa byesi bakhulile ukhwana khuiduka oba mukhatale? | 1. [_] Eeh  2. [_] Taawe |  | EH: RB  VN: 1Yrn05 |
| 6. When do you usually wash hands? | 1. [_] Before cooking  2. [_] Before eating  3. [_] Before feeding the child  4. [_] after visiting a toilet | 6. Osingasakha liina kimikhono kyowo? | 1. [_] Nga oshili khutekha  2. [_] Nga oshili khulya  3. [_] Nga oshili khuliisa umwana  4. [_] Nga waamile mushikho | **RULE: Tick off all that apply** | EH:CB  VN:1Yrn06 |
| 7. When do you usually wash the child’s hands? | 1. [_] Before feeding the child  2. [_] after visiting a toilet | 7. Osingasakha liina kimikhono kyo omwana? | 1. [_] Nga oshili khuliisa umwana  *2. [_] Nga wamile mu shiko* | **RULE: Tick off all that apply** | EH: CB  VN:1Yrn07 |
| 8. Do you usually wash  your hands with soap? | 1. [_] Yes  2. [_] No | 8. Usingasaka kimikhono kyowo ni sabuni? | 1. [_] Eeh  2. [_] Taawe |  | EH: RB  VN:1Yrn08 |
| 9. Do you usually wash <NAME’s> hands with soap? | 1. [_] Yes  2. [_] No | 9. Usingasaka kimikhono kya <LIISINA> ni sabuni? | 1. [_] Eeh  2. [_] Taawe |  | EH: RB  VN:1Yrn09 |
| 10. Do you cook in advance for <NAME> and store leftovers? | 1. [_] Yes  2. [_] No | 10. Utekhela <LIISINA> nga bushili wamala wabikha biramile? | 1. [_] Eeh  2. [_] Taawe | **If No, skip to question 12** | EH: RB  VN:1Yrn10 |
| 11. Do you re-warm stored food thoroughly before serving it to the child | 1. [_] Yes  2. [_] No | 11. Ukhasakho bilyo byesi obikhile umwana bulayi bwene nga oshili khumuwa | 1. [_] Eeh  2. [_] Taawe | **May need to ask how long it takes to reheat** | EH:RB  VN:1Yrn11 |
| 12. Is <NAME> able to feed himself / herself? | 1. [_] Yes  2. [_] No | 12. <LIISINA> anyala ukhuiliisa yenyene? | 1. [_] Eeh  2. [_] Taawe | **If yes, skip to 14** | EH:RB VN:1Yrn12 |
| 13. If not, how do you help <NAME > eat? | ______________ | 13. Nga taawe, oyeta uryena <LIISINA> khulya? | **__________________** |  | EH:Tx VN:1Yrn13 |
| 14. Who generally feeds <NAME>? | 1. [_] Yourself  2. [_] Sibling  3. [_] Father  4. [_] Neighbour  5. [_] Relatives /friends  6. [_] Other specify_________ | 14. Khuyilila atwera, nanu uliisa <LIISINA>? | 1. [_] Wamwene  2. [_] Yaya weye  3. [_] Papa weye  4. [_] umulilwana  5. [_] Balebe beye/basale  6. [_] Bandi shiina,baboole__________ |  | EH:RB  VN:1Yrn14 |
| 15. How old is this person? | _________ | 16. Umundu uyu alinabukhulu shiina? | **____________________** |  | EH:Num  VN:1Yrn15 |
| 16. What if this person is not available? | 1. [_] No one  2. [_] Yourself  3. [_] Sibling  4. [_] Father  5. [_] Neighbour  6. [_] Relatives /friends  7. [_] Other specify_________ | 16. Ne nga umundu uyu saliwo ta? | 1. [_] Mbawo umundu yesi  2. [_] Wamwene  3. [_] Yaya weye  4. [_] Papa weye  5. [_] Balebe beye/basale  6. [_] Bandi shiina,baboole__________ |  | EH:RB VN1Yrn16 |
| 17. Does <NAME> have his or her own bowl? | 1. [_] Yes  2. [_] No | 17. <LIISINA> ali nisowani yewe? | 1. [_] Eeh  2. [_] Taawe |  | EH:RB VN1Yrn17 |
| 18. Do you sit down with <NAME> when he ir she is eating? | 1. [_] Yes  2. [_] No | 18. Wikhala asi ni <LIISINA> nga alikhulya? | 1. [_] Eeh  2. [_] Taawe |  | EH:RB VN1Yrn17 |

## VI Anthropometry

**EpiHandy p. m**

| 1. Baby’s weight | _____________kg (#.#) |  |  | EH: Num  VN:24m01 |
| --- | --- | --- | --- | --- |
| 2. Baby’s length ______ | _____________cm (##.#) |  |  | EH: Num  VN: 24m02 |
| 3: Other comments |  |  |  | EH: Large text field  VN: 24m03 |
