# Supplementary material for: Effects of complementary feeding on attained height among lower primary school-aged children in Eastern Uganda: A nested prospective cohort study
Source: PLoS One. 2019 Feb 7;14(2):e0211411. doi: 10.1371/journal.pone.0211411 (PMC6366764; doi:10.1371/journal.pone.0211411)
Supplement: S1 Table — (DOCX) [file pone.0211411.s005.docx]

**S1 Table. Unadjusted and multivariable linear regression analyses of the association between the contextualized adequate CF at 18-24 months of age and attained height (HAZ) at age 5-8 years, without HAZ and WHZ at baseline**

| Covariate | Unadjusted slope (95% CI) | P-value | Adjusted slope (95% CI) | P-value |
| --- | --- | --- | --- | --- |
| CF |  |  |  |  |
| Inadequate | Ref. |  | Ref. |  |
| Adequate at 18-24 months | 0.032 (-0.261, 0.326) | 0.822 | -0.111 (-0.363, 0.141) | 0.374 |
|  |  |  |  |  |
| Child age at 5-8 years visit | -0.299 (-0.465, -0.132) | 0.001 | -0.244(-0.395,-0.092) | 0.003 |
|  |  |  |  |  |
| Residence |  |  |  |  |
| Urban | Ref. |  | Ref. |  |
| Rural | -0.670 (-1.204, -0.136) | <0.016 | -0.506(-0.987,-0.025) | 0.040 |
|  |  |  |  |  |
| Wealth status |  |  |  |  |
| Poor | Ref. |  | Ref. |  |
| Middle | 0.095 (-0.165, 0.354) | 0.458 | 0.046 (-0.246, 0.338) | 0.747 |
| Least poor | 0.577 (0.190, 0.964) | <0.005 | 0.273 (-0.080, 0.625) | 0.123 |
|  |  |  |  |  |
| Mother’s education (years) | 0.057 (0.0193, 0.095) | 0.005 | 0.033 (0.003, 0.063) | 0.031 |
|  |  |  |  |  |
| EBF status |  |  |  |  |
| No | Ref. |  | Ref. |  |
| Yes | -0.292 (-0.549, -0.034) | 0.028 | -0.215 (-0.469, 0.038) | 0.092 |
|  |  |  |  |  |
| Electricity |  |  |  |  |
| No | Ref. |  |  |  |
| Yes | 0.218 (-.020, 0.456) | 0.071 | 0.050 (-0.180, 0.280) | 0.656 |
|  |  |  |  |  |
| ANC attendance |  |  |  |  |
| No | Ref. |  |  |  |
| Yes | 0.237 (0.023, 0.451) | 0.031 | 0.164 (-0.021, 0.349) | 0.080 |
|  |  |  |  |  |
| Marital status of the parents |  |  |  |  |
| Married | Ref. |  |  |  |
| Cohabiting | 0.117 (-0.154, 0.389) | 0.381 |  |  |
| Not in union | -0.310 (-0.721, 0.101) | 0.133 |  |  |
|  |  |  |  |  |
|  |  |  |  |  |
|  |  |  |  |  |
| Father’s education (years) | 0.036 (0.001, 0.080) | 0.113 |  |  |
|  |  |  |  |  |
| BF at 18-24 months |  |  |  |  |
| No | Ref. |  |  |  |
| Yes | 0.108 (-0.102, 0.319) | 0.298 |  |  |
|  |  |  |  |  |
| Parity |  |  |  |  |
| Primipara | Ref. |  |  |  |
| Multipara | -0.048 (-0.385, 0.290) | 0.773 |  |  |
|  |  |  |  |  |
| Water source |  |  |  |  |
| Surface water or other | Ref. |  |  |  |
| Borehole or tap | 0.094 (-0.182, 0.370) | 0.487 |  |  |
| Piped yard or home | 0.235 (-0.343, 0.813) | 0.409 |  |  |

Ref.: comparison category. Regression modelling estimates are based on heteroscedasticity-robust standard errors. BF: Breastfeeding.
